# Functional Gene Expression Differentiation of the Notch Signaling Pathway in Female Reproductive Tract Tissues—A Comprehensive Review With Analysis

**DOI:** 10.3389/fcell.2020.592616

**Published:** 2020-12-15

**Authors:** Magdalena Orzechowska, Dorota Anusewicz, Andrzej K. Bednarek

**Affiliations:** Department of Molecular Carcinogenesis, Medical University of Lodz, Lodz, Poland

**Keywords:** female tract, notch signaling, carcinogenesis, breast cancer, ovarian cancer, endometrial cancer, cervical cancer

## Abstract

The Notch pathway involves evolutionarily conserved signaling regulating the development of the female tract organs such as breast, ovary, cervix, and uterine endometrium. A great number of studies revealed Notch aberrancies in association with their carcinogenesis and disease progression, the management of which is still challenging. The present study is a comprehensive review of the available literature on Notch signaling during the normal development and carcinogenesis of the female tract organs. The review has been enriched with our analyses of the TCGA data including breast, cervical, ovarian, and endometrial carcinomas concerning the effects of Notch signaling at two levels: the core components and downstream effectors, hence filling the lack of global overview of Notch-driven carcinogenesis and disease progression. Phenotype heterogeneity regarding Notch signaling was projected in two uniform manifold approximation and projection algorithm dimensions, preceded by the principal component analysis step reducing the data burden. Additionally, overall and disease-free survival analyses were performed with the optimal cutpoint determination by Evaluate Cutpoints software to establish the character of particular Notch components in tumorigenesis. In addition to the review, we demonstrated separate models of the examined cancers of the Notch pathway and its targets, although expression profiles of all normal tissues were much more similar to each other than to its cancerous compartments. Such Notch-driven cancerous differentiation resulted in a case of opposite association with DFS and OS. As a consequence, target genes also show very distinct profiles including genes associated with cell proliferation and differentiation, energy metabolism, or the EMT. In conclusion, the observed Notch associations with the female tract malignancies resulted from differential expression of target genes. This may influence a future analysis to search for new therapeutic targets based on specific Notch pathway profiles.

## Introduction

With a growing global burden, the prevention and management of female cancers remain challenging. Breast cancer (BC) accounted for a quarter of newly diagnosed cases followed by cervix uteri (CC), uterus corpus endometrial (EC), and ovarian carcinomas (OV) contributing 6.9, 5.3, and 3.6% of the total number of new cases diagnosed in 2018, respectively (Bray et al., 2018).

The female tract comprises internal and external organs that together form a system working in complexity to carry out several functions, basically related to reproduction. Regarding the importance of their mission, maintaining the homeostasis of these tissues seems challenging as well as extremely significant. Any unbidden deregulation of the homeostasis may result in poor outcomes, e.g., gynecological (including endometrial, cervical, and ovarian cancers) as well as breast malignancies (Bates and Bowling, 2013).

The Notch pathway is one of the key regulators in the development of breast, cervix, ovary, and uterine endometrium epithelial tissues and is commonly affected during carcinogenesis and cancer progression (Mitsuhashi et al., 2012; Groeneweg et al., 2014; Kontomanolis et al., 2018; Rodrigues et al., 2019). In the present essay, we comprehensively review Notch-driven gene expression differentiation of specific tissues that are simultaneously dependent on signaling by steroid hormones. Further, we discuss the alterations of Notch signaling at two levels of action: the canonical core signaling and downstream effects of signal transduction in the context of female tract tumorigenesis and cancer progression. We additionally enriched the current review with our new analyses involving The Cancer Genome Atlas (TCGA) expression data to present the complex view of Notch-driven carcinogenesis in hormone-dependent female tract tissues. We address the question of how does the Notch signaling orchestrate cellular differentiation and proliferation within the normal breast, ovarian, uterus endometrial, and cervical tissues in comparison with cancerous tissues, especially in the context of steroid hormone dependency.

Notch signaling is a highly conserved pathway that originated from genetic studies in Drosophila melanogaster, specifically from observations of mutant flies with notched wings (Dexter, 1914). This signaling mechanism stands out as a crucial player in the transmission of internal information, thus governing many processes at different stages of development from cell fate determination during embryogenesis to differentiation, growth, and apoptosis control in postnatal life (Artavanis-Tsakonas et al., 1999).

Regarding Notch's importance in multicellular organisms, it is surprisingly simple in molecular design, containing a relatively small number of canonical core members. In humans, there are four Notch receptors (Notch1–4) and five canonical ligands belonging to the Delta–Serrate–Lag (DSL) family (Jag1, Jag2, Dll1, Dll3, and Dll4) (D'Souza et al., 2010). In canonical signaling, transmembrane Notch receptor interacts by its extracellular domain with one DSL ligand on a neighboring cell and initiates a sequence of two proteolytic cleavage events: first, catalyzed by tumor necrosis factor α-converting enzyme (TACE), viz., disintegrin-metalloproteinase of ADAM family (Adam10, Adam17), and second, by intracellular γ-secretase complex (comprising Psen1, Psen2, Pen2, Aph1, and nicastrin) resulting in the release of the Notch intracellular domain (NICD). Processing of Notch receptors involves posttranslational fucosylation by O-fucosyltransferase 1 (Pofut1) in endoplasmic reticulum followed by further modifications carried out by lunatic (Lfng), manic (Mfng), or radical fringe (Rfng) that occurs in the Golgi and regulates interactions with ligands (Logeat et al., 1998). Moreover, interactions of an activation nature between extracellular domains of Notch receptors and ligands appear in the form of trans-activation between juxtaposed cells, whereas cis-inhibition blocks interactions between proteins co-expressed along the membrane of the same cell (Saxena et al., 2001). Afterward, NICD translocates to the nucleus, interacts with CBF-1/Suppressor of Hairless/Lag-1 DNA-binding protein (CSL), also known as recombination signal binding protein RBP-J, and, with the addition of mastermind-like 1 (Maml1), forms a trimeric coactivator complex leading to expression of Notch direct executives of the HES/HEY family (Hes1, Hes5, Hey1, Hey2, and HeyL) forwarding the signal downwards to the final effectors (Andersson et al., 2011). Both HES and HEY are to date the best-known mammalian representatives of primary CSL-related Notch signaling targets belonging to the basic helix-loop-helix (bHLH) family of transcription factors (TFs), acting mainly as gene transcription repressors (Fischer and Gessler, 2007). A wide variety of cellular processes and events that Notch signaling governs through HES/HEY may therefore be explained by a great abundance of targeted genes. Beside HES and HEY, additional direct targets of Notch have also been reported; some of them depend on Notch signals in multiple tissues, while others are limited to specific types, including, e.g., pivotal cell cycle regulators (e.g., p21, p27, and cyclin D1), growth factors (e.g., ErbB2), regulators of apoptosis, and other TFs (e.g., c-Myc and NF-κB) (Miele and Osborne, 1999; Miele, 2006; Miele et al., 2006). Notch signaling can also be initiated in a non-canonical, i.e., ligand-independent manner as has been identified mostly in undifferentiated cell populations (Deftos et al., 2000). To date, three types of non-canonical Notch signaling have been distinguished: Type I—CSL-independent, Type II—S3 cleavage-independent, and Type III—Notch cleavage- and NICD release-independent (Sanalkumar et al., 2010). Worth noting is the fact that thus Notch signaling may be activated independently of NICD formation, which suggests cross-talk of the Notch pathway upstream of NICD processing.

Remarkable Notch pleiotropy of its transcriptional output is a corollary to a regulation pattern that Notch undergoes through pre-existing states of chromatin set by upstream “pioneer” TFs. For instance, Ditadi et al. demonstrated that differentiation of adult-type hematopoietic cells in the dorsal aorto-gonadal-mesonephros (AGM) region is indeed dependent on Notch1 and, more importantly, placed Notch upstream of pioneer TFs such as Runx1, Myb, and Gata2 during this process. It suggests that at point of lineage decision branch, Notch may act in combination with particular pioneer TFs that activate secondary downstream TFs, which subsequently modify chromatin landscape to allow Notch initiation of different transcriptional output preceding downstream cell fate decisions (Ditadi et al., 2015). It was shown that among pre-existing cellular potentials, Notch specifies cell fate commitment through two classical modes: first, lateral inhibition, and second, lateral induction. By the former mechanism, cells adopt a particular fate and simultaneously inhibit adjacent cells from developing in a similar manner. Conversely, the latter implies sustaining a particular state of cell or group of cells that leads surrounding cells to differentiate (Flores et al., 2000; Haines and Irvine, 2003). Besides, cell fate determination was linked to the asymmetry occurring between Jagged and Delta ligands that gives rise to cells in a specific possible state: Sender, Receiver, or Sender/Receiver hybrid phenotype. The Notch-Delta signaling represents the mechanism of lateral inhibition leading to the acquisition of the opposite fates by the two cells, where the first cell shows high ligand (Delta) and low receptor (Notch) expression on its surface, whereas the second cell shows low ligand (Delta) and high receptor (Notch) expression. Hereby, the first cell serves as a Sender and the other cell serves as a Receiver. In contrast, the Notch-Jagged signaling is an example of the lateral induction resulting in the acquisition of a similar fate by the two cells, where both cells have an intermediate expression level of both the receptors (Notch) and ligands (Jagged); therefore, both may act as Sender and Receiver (Sender/Receiver hybrid phenotype). Despite lateral inhibition, as well as lateral induction, occurring in physiology (e.g., neurogenesis control in vertebrates; Beatus and Lendahl, 1998 in the former and mammalian inner-ear development; Hartman et al., 2010 in the latter), it has also been associated with pathology such as tumor–stroma cross-talk frequently involving Notch-Jagged signaling (Boareto et al., 2015b). Furthermore, in contrast to other pathways, Notch does not involve secondary messengers to amplify the signals and is solely dependent on the nuclear concentration of NICD (Kovall, 2007). Each activated receptor molecule is being consumed, which yields one NICD, indicating a strict association of signaling input and output, making signal strength essential for eliciting a specific cellular response but, on the other hand, sensitizing Notch to even small deviations from baseline expression (Fanto and Mlodzik, 1999). Hence, relatively weak and short Notch signals may activate only some subset of targets genes, while stronger signals of longer duration (as, e.g., in tumor cells with the constitutively active Notch pathway) may activate larger extent of target genes and even govern genes that primarily remain out of Notch regulation at physiological doses (Aster et al., 2017). Hereby, Notch signaling becomes even more significant; its sensitivity to alterations in expression together with a diverse repertoire of supervised biological processes draws a clear conclusion that any deregulation may lead to severe disruption of a particular mechanism and a further perspective to carcinogenesis.

### Notch in Tumorigenesis

**Figure 1:**
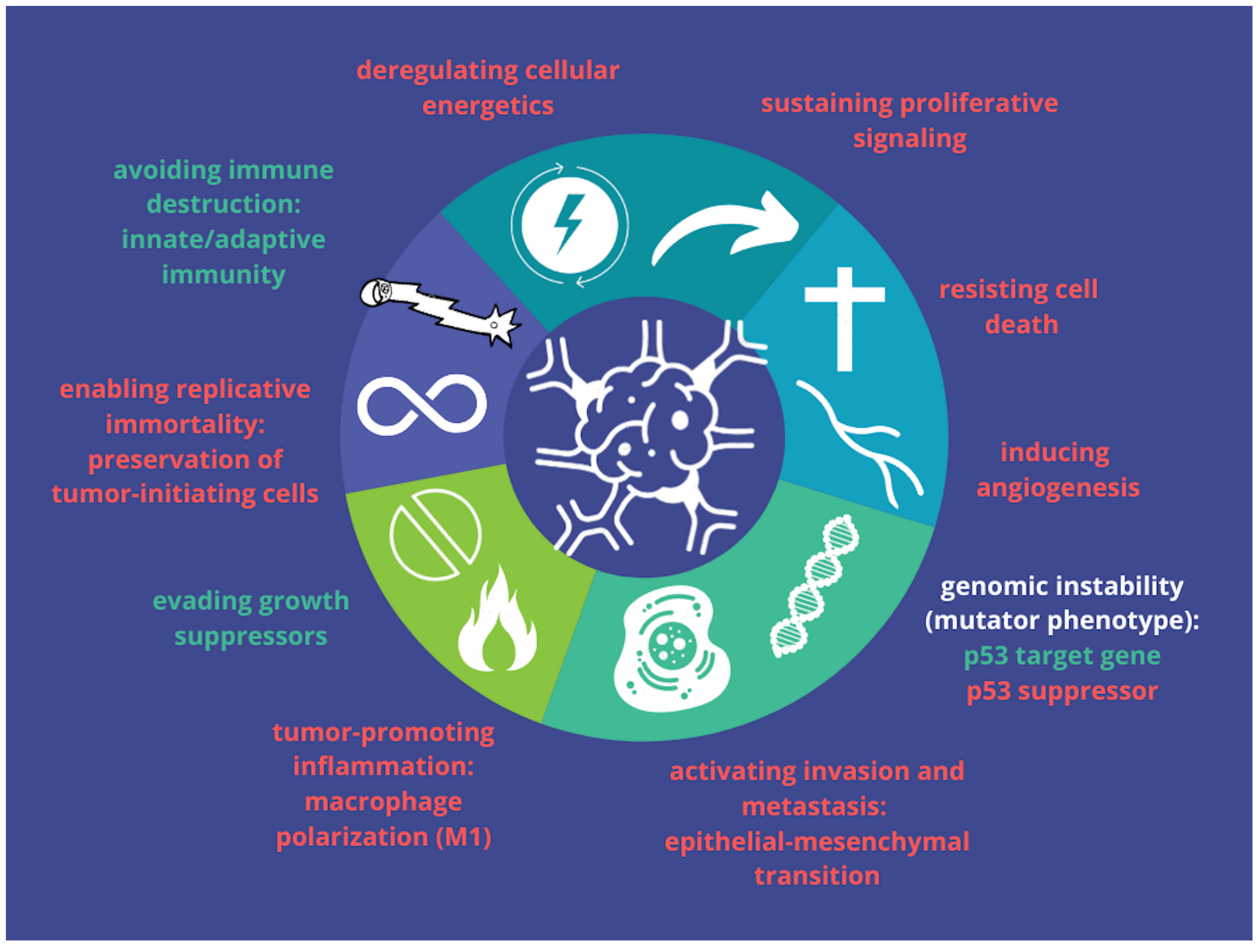
Symbolic representation of cancer hallmarks that are proposed to be affected by Notch signaling. Oncogenic effects are shown in red and tumor-suppressive effects are in green (based on Aster et al., 2017).

Regarding paradoxical roles that Notch plays during development, either block or promotion of differentiation in a cell type/fate-dependent manner, both hyper- and hypoactivation of the pathway can lead to tumor formation and progression. Remarkably, effects of Notch deregulation, same as cellular outcomes, are tissue- and, therefore, cancer-specific and reflect the diverse roles of Notch in a different context in cancers. An emerging body of evidence revealed Notch implications in all fundamental hallmarks of cancer demonstrated by Hanahan and Weinberg (2000, 2011), which range from oncogenic to tumor suppressive dependent on cancer type and tissue of origin as well as a set of downstream effectors that are turned on or off (Radtke and Raj, 2003; Nowell and Radtke, 2017b) (Figure 1). Moreover, the Notch pathway belongs to the group of cell fate arbiters, which regulates the balance between differentiation and division. Vogelstein et al. in the review of cancer genome landscapes pointed selective growth advantage of cancerous cells due to favoring the latter process through Notch abrogation (Vogelstein et al., 2013).

Notch was for the first time linked to tumorigenesis in human T-cell acute lymphoblastic leukemia (T-ALL) through the identification of chromosomal translocation [t(7:9)(q34;q34.3)], resulting in the juxtaposition of Notch1 and T-cell receptor β promoter (TCF-β) truncating Notch1 (Ellisen et al., 1991, p. 1). Following this finding, Notch alterations have been reported in numerous tumors including solid and hematological malignancies. Table 1 illustrates the Notch roles in exampling malignancies.

**Table 1:** Several examples reflecting diverse roles of Notch pathway in a cell- and cancer-specific manner.

**Function of notch in**	**References**
**Oncogenic**	
Blood	Koch and Radtke, 2011a,b
Brain	Fan et al., 2004, p. 2; Purow et al., 2005; Gao et al., 2007; Kanamori et al., 2007; Zhang X.-P. et al., 2008; Gaetani et al., 2010; Zhao et al., 2010
Lungs	Zheng et al., 2013
Breast	Pece et al., 2004; Reedijk et al., 2005; Ayyanan et al., 2006; Hu et al., 2006; Sansone et al., 2007; Shipitsin et al., 2007
Pancreas	Miyamoto et al., 2003; De La O et al., 2008; Plentz et al., 2009; Mazur et al., 2010; Maniati et al., 2011; Cook et al., 2012
**Tumor suppressive**	
Skin	Nicolas et al., 2003; Wang et al., 2011; Pickering et al., 2014
Esophagus	Alcolea et al., 2014; Gao et al., 2014; Song et al., 2014
Lungs	George et al., 2015
Liver	Viatour et al., 2011
Pancreas	Hanlon et al., 2010

To date, the best-known oncogenic activity of Notch in human malignancies is heightening the expression of pro-growth and proliferative genes. Research supporting this phenomenon was in major part conducted in human and murine T-ALL *in vitro* and *in vivo* models and focused on Notch ability to increase the expression of one of the global regulators of growth metabolism—MYC (Sharma et al., 2006; Weng et al., 2006; Palomero et al., 2007; Dang et al., 2009). Besides, Swamy et al. demonstrated that Notch promotes the O-GlcNAcylation of proteins, which is dependent on a constant influx of glucose and glutamine. One of the proteins that is modified through this process is Myc, which suggests its potential role as a sensor of nutrient sufficiency downstream of Notch signaling promoting the further progression of the cell cycle (Swamy et al., 2016). Another study showed cross-talk between the Notch and the PI3K-Akt pathway that may enhance the Warburg effect through increasing expression of glucose transporters by Akt (Palomero et al., 2007). Additionally, Notch was also shown to interact with the hypoxia pathway through hydroxylate hypoxia-inducible factor (HIF). It was proposed to integrate hypoxia with epithelial-to-mesenchymal transition (EMT) in tumor cells since the Notch/hypoxia axis was presented in the control of stem cell (SC) differentiation (Gustafsson et al., 2005; Sahlgren et al., 2008). Notch itself was shown to be a key initiator of EMT (Timmerman et al., 2004; Zavadil et al., 2004; Niessen et al., 2008; Sahlgren et al., 2008). Specifically, it was suggested that Jag1 mediates activation of Notch signaling during triggering EMT in epithelial cells (Noseda et al., 2004). Another study revealed a direct interaction between Notch1 and Snail-1, yielding the downregulation of VE-cadherin and loss of contact inhibition *in vitro* (Timmerman et al., 2004). Notch has also been correlated with activation of NF-κB, another prosurvival TF; however, to date, the mechanism remains elusive. Espinosa et al. demonstrated that HES1 suppresses the expression of Cyld, a known inhibitor of NF-κB, thus leading to hyperactivation of NF-κB signaling and enhanced survival of T-ALL cells *in vitro* (Espinosa et al., 2010, p. 1). Finally, other oncogenic mechanisms that are employed by Notch include inhibition of apoptosis through downregulation of proapoptotic TF, Nur77, upregulation of Bcl2, IAP, and FLIP as well as inhibition of JNK activation.

On the other hand, Notch presents a tumor-suppressive character that was reported in several malignancies of squamous cell types such as head and neck, cutaneous, lung, bladder, and esophageal carcinomas and manifested itself through mutations predominantly found in Notch receptors (NOTCH1–4). Additionally, other alterations that reduced Notch activity were reported, e.g., loss-of-function mutations detected in Notch members like MAML1 and JAG2, and importantly, all these findings were confirmed in numerous *in vivo* studies employing murine models (Nowell and Radtke, 2017a). Leong et al. proposed another suppressive mechanism that Notch may be involved in inhibition of proliferation and induction of cell cycle arrest through increased expression of p21^Cip1^ and p27^Kip1^ as well as decreased β-catenin-mediated Wnt signaling (Leong and Karsan, 2006). Not only truncation or downregulation of Notch may have tumor-suppressive effects. Surprisingly, it was reported that the constitutive activation of Notch1 may suppress cellular growth in HPV-positive cervical cancer cells. The proposed mechanism comprises repression of viral E6/E7 expression by Notch through AP-1 downregulation that leads to increased p53 expression and prevents hyperphosphorylation of pRb. Alternatively, activation of Notch1 suppresses expression of E47, a helix-loop-helix TF, through ERK1/2 activation, hence inhibiting the progression of the cell cycle (Talora et al., 2002, 2005). A corollary to the above is a conclusion that a decrease in Notch1 activity appears to be more important during late tumorigenesis, rather than early tumor formation stage. Also, different Notch receptors may even have opposing effects within the same malignancy. In particular, Notch1 and Notch2 were shown to have antagonistic effects in embryonal brain tumor cell lines, where it went out that Notch2 promoted while Notch1 inhibited cell proliferation, soft agar colony formation, as well as xenograft growth (Fan et al., 2004, p. 2). In summary, it becomes apparent that Notch has dualistic character during carcinogenesis, from oncogenic to tumor suppressive, which seems to be dependent on the cellular context and Notch cross-talk with other signaling pathways, although the protective character of Notch remains less well-understood than oncogenic and remains to be further investigated.

### Notch in the Normal Development

#### Breast

Murine models have been found to be very useful in studies on mammary gland development and its tumoric transformation. The physiological development of murine mammary glands involves sequential steps of proliferation, morphogenesis, and differentiation events that ultimately lead to the formation of the epithelial duct system (Daniel and Smith, 1999). A large part of growth-associated and developmental processes occurs after birth and progresses among defined stages of puberty and pregnancy, ultimately leading to initiation of lactation (the above processes have been described in greater detail elsewhere; Smith and Boulanger, 2003). The remarkable essence in the context of mammary gland development is the interaction of multiple kinds, mesenchymal–epithelial, between epithelial, and involving the extracellular matrix (ECM), which are accompanied by apoptosis during involution of mammary gland after the lactation period. As expected, regarding the decisive role of Notch in determining cell fate, canonical signaling has been shown in several studies as an essential regulator of mammary cell communication during embryogenesis, SC self-renewal, cell lineage commitment, proliferation, and differentiation, as well as apoptosis in both murine and human mammary glands (Harrison et al., 2010; Takebe et al., 2011). Raafat et al. demonstrated temporal and spatial regulation of Notch in epithelial cells of mammary glands during development *in vivo*. In the adult tissues, NOTCH1–3 expression was increased from 5 weeks of age through early pregnancy onset followed by decrease observable with more advanced pregnancy stages and mammary gland involution after lactation (both apoptotic and quiescent mammary glands). Regarding receptors, NOTCH3 was the most abundant among all developmental stages in contrast to NOTCH4, whose expression was undetectable. Among other members of the Notch core, JAG1, DLL3, and HEY2 showed the highest expression among ligands and family of Hes/Hey genes analyzed during different stages of postnatal mammary gland development, respectively (Raafat et al., 2011). In turn, constitutively active NOTCH4 (Int3) controlled by mouse mammary tumor virus (MMTV) promoter in transgenic mice affected cell fate selection in the mammary epithelial cells, particularly arresting mammary gland development with a reduction in ductal growth and secretory lobule development that eventually led to the loss of lactation followed by transformation into poorly differentiated adenocarcinomas (Jhappan et al., 1992). Results referring to NOTCH4 have been confirmed in previously conducted studies involving the same transgenic model. In 2000, Soriano et al. proposed Notch4 as an oncoprotein presenting its constitutive activity in mammary glands, failing in the development of secretory lobules during gestation with further transformation in mammary tumors, hence making both findings consistent (Soriano et al., 2000).

In humans, breast tissue varies with the cyclic period throughout a woman's life. Puberty is characterized by the onset of the very rapid growth of breast accompanied by the expansion of blunt-ended primary and secondary ducts that ultimately branch into a complex tree with terminal ductal/lobular-alveolar units (TDLUs). The subsequent period between menarche and menopause exposes breasts to significant fluctuations in growth according to the clock of menstrual cycles. In turn, during pregnancy, the mammary gland is being extensively prepared for lactation through side branching and alveolar development. Subsequent cessation of milk production and involution represses the previous state, thereby resembling similarity to the virgin mammary gland. Such constant changes suggested the potential existence of mammary SCs (Williams and Daniel, 1983), which to date have been broadly studied and described (e.g., Kordon and Smith, 1998; Dontu et al., 2003; Shackleton et al., 2006; Stingl et al., 2006).

Recently, the epithelium of normal mammary gland has emerged in a form of a mixture of differentiated cell populations arranged in a hierarchical pattern with their stem and progenitor cells that are controlled by evolutionary pathways determining cell fate such as unsurprisingly, Notch signaling. Dontu et al. implicated Notch in self-renewal of the normal mammary SCs as well as progenitor compartments *in vitro* by applying a multicellular spheroids system for culturing putative mammary SCs (so-called “mammospheres”). This study indicated that the mammospheres' ability of self-renewal (equated with SC self-renewal capacity) was significantly increased in cultures enriched with a synthetic peptide derived from the Delta–Serrate–Lag2 (DSL) domain capable to activate the Notch pathway (Dontu et al., 2003, 2004). Another study investigated the role of Notch in mammary SCs by applying different *in vitro* culturing systems consisting of genetically manipulated epithelial subpopulations followed by testing their repopulating abilities in the cleared mammary fat pad of mice. Worth noting, it was concluded that Notch is required to repopulate precursor populations at the early stages of establishing the hierarchy in the mammary epithelium (Bouras et al., 2008). Moreover, as reviewed by Melchor and Smalley, among different human mammary cell populations, genes involved in core Notch signaling exhibited differential expression patterns between two specific populations characterized by different colony-forming capacities: bipotent colony-forming cells (CFCs) and luminal-restricted CFCs. In particular, upregulation and downregulation of NOTCH4 was identified in the former and latter cells, respectively, in opposition to remaining Notch receptors (NOTCH1–3) as well as HES6. Further studies on NOTCH3 led to the conclusion that it may be considered as a key gene for the luminal cell commitment; although it was not explicitly stated, bipotent CFCs could correspond to stem progenitor cells, whereas the luminal CFCs may be considered as a linage-restricted progenitor population (Melchor and Smalley, 2008). In addition, distinct profiles of Notch1 expression were identified among different subtypes with remarkably high expression in the luminal-type cells (Bouras et al., 2008; Rodilla et al., 2015). Ultimate downregulation of Cbf-1/RBP-jk affected absolute SC number since it increased proliferation rate in SCs, although such an increase in proliferation had false bottom manifesting in disorganized side branching with a shifted contribution toward basal-type cells in the end buds and thus regulating the formation of more basal cell phenotypes. Similar effects were observed with overexpression of Numb, which is an endocytic negative regulator of Notch. In contrast, Notch1 upregulation was associated with commitment to the luminal cell lineage (more precisely: high keratin 8/18, Stat5, and p63 downregulation) (Bouras et al., 2008). Recently, *in vivo* imaging revealed basal SCs in the mammary gland of bipotent character that could yield in both myoepithelial and luminal cells (Rios et al., 2014) and Notch was found in charge during this process (Tiede and Kang, 2011; Junankar et al., 2015; Rodilla et al., 2015; Pamarthy et al., 2016).

Discovery of SCs entails the theory of tumor-initiating cells [TICs, also known as cancer stem cells (CSCs); both terms are used interchangeably] of large tumorigenic potential that drives carcinogenesis (Al-Hajj et al., 2003; Fu et al., 2014). By analogy to somatic SCs in organogenesis, tumors are composed of multiple cell types framed in a hierarchical pattern beginning with TICs that possess self-renewal capacity to repopulate the tumor. In breast carcinomas, TICs were initially characterized as lineage-negative (lin-) CD44+/CD24-/low cells (Al-Hajj et al., 2003). By applying the previously conceived methodology of culturing SCs in mammospheres, Ponti et al. cultured putative breast TICs *in vitro* in multicellular tumorspheres. As was demonstrated, tumorspheres, similarly to mammospheres, consist of undifferentiated cells able to self-renew and create another generation of tumorspheres involving cells differentiating into ductal and myoepithelial mammary lineages (Ponti et al., 2005). Investigations focusing on Notch signaling in tumorspheres derived from ductal carcinoma *in situ* (DCIS) showed a significant reduction of mammosphere production when the signaling was inhibited by either γ-secretase inhibitors (GSI), an anti-Notch4 monoclonal antibody (mAb), or gefitinib, the anti-EGFR compound, thereby suggesting indispensability of Notch for expansion of TICs in DCIS (Farnie et al., 2007). Moreover, these findings shed light on EGFR and Notch cooperation in TICs biology, which complies with regulatory feedback loop involving Notch and Her2 possibly maintaining TICs in HER2-enriched BCs (Korkaya and Wicha, 2009).

As described later in the section devoted to BC characteristics, it comprises a heterogeneous collection of molecular subtypes that differ in prognosis and available treatment options. To date, several studies suggested Notch activation in association with particular BC subtypes, especially triple-negative BC (TNBC). Although the relevance of Notch and how it influences the development of particular BC subtype are still elusive, the main explanation refers to its well-established role in SC lineage specification that was for the first time proposed in 2006 by Buono et al. Based on the Cre-mediated deletion model, the authors identified Notch maintaining luminal cell fate to the detriment of uncontrolled basal cell proliferation during alveolar development (Buono et al., 2006). To support the above hypothesis, another research revealed the indispensability of Notch3 during the commitment of bipotent progenitors to the luminal lineage (Raouf et al., 2008). Together with the aforementioned investigations of Bouras et al., the role of Notch in the expansion of the luminal progenitor population in the mammary glands became apparent. Furthermore, to explain the specific association of Notch in origins of TNBC, another model was suggested whereby aberrant Notch signaling contributes to the expansion of abnormal luminal progenitor population that ultimately initiate basal-like carcinoma; however, the model was only shown in carriers of BRCA1 mutation (Lim et al., 2009).

#### Ovaries, Endometrial Epithelium of the Uterus, Cervix, and Endocervix

Notch signaling is one of the most conserved developmental pathways in multicellular organisms such as mammals. Establishing its role in the development of the female reproductive system, i.e., ovaries, uterine endometrium, and cervix, is currently a major focus of multiple research. Even though the role of Notch in the development of these organs was very well-determined in model organisms including *D. melanogaster* and *C. elegans* (Andersson et al., 2011; Greenwald and Kovall, 2013), the insight into its function in normal gonads is very narrow, conversely to tumorigenesis.

Ovarian morphogenesis in mammals is a process that requires very precise spatial and temporal coordination of functions involving multiple types of cells, which is achieved by the mechanisms of endo-, para-, auto-, and juxtacrine signaling. The last type of signaling is remarkably executed by Notch as a contact-dependent pathway.

To date, Notch was revealed in both the embryonic and postnatal ovarian development, especially in essential events including follicle assembly and growth, meiotic maturation, vasculogenesis of ovaries, and production of steroid hormones. Importantly, NOTCH2, JAG1, JAG2, HES1, and HEY2 were the most abundantly expressed among all Notch core members within embryonic ovaries (reviewed in Vanorny and Mayo, 2017).

To date, multiple evidence indicated the role of Notch in the development of ovaries in mammals. For instance, Vanorny et al. presented a model in which overexpression of JAG1 and JAG2 in the oocyte signals through NOTCH2 that is present among pregranulosa cells to take a part in the formation of germ cell syncytia and assembly of primordial follicles (Vanorny et al., 2014). On the other hand, studies inhibiting Notch signaling with either GSI or RNAi revealed disruption of multiple developmental processes ongoing in ovaries, hence indicating Notch involvement in meiotic progression and follicle assembly. Feng et al. demonstrated consequences of knockdown of NOTCH1 including delayed meiotic progression, defective oocyte growth, and aberrant primordial follicle assembly followed by the formation of multi-oocyte follicles within renal grafts of embryonic ovarian tissues (Feng et al., 2014, 2016). By employing an *ex vivo* ovarian culture system, it was shown that Notch inhibition delays syncytial breakdown, decreases granulosa cell proliferation, and grows the pool of faulty oocytes due to formation of the abrogated follicular niche (Trombly et al., 2009; Chen et al., 2014; Terauchi et al., 2016). Additional research revealed the emerging role of Notch in the development of mammalian ovaries involving the promotion of the growth and maturation of ovarian follicles through interactions between juxtaposed follicular cells as well as other interactions between cells of different types that require Notch signaling for proper luteinization and vasculature of the ovaries. It was shown that productive Notch signaling is an essential element for the local microenvironment, where the female germ cell develops distinct roles throughout developing ovarian follicles to ensure basic female reproductive functions (Vanorny and Mayo, 2017). Worth noting, multiple observations indicated that proper maintenance of Notch signaling requires an appropriate steroid hormone environment, thus confirming Notch cross-talk with steroid hormone signaling in both *in vitro* and *in vivo* conditions (Guo et al., 2012, 2).

In contrast to mammalian models, the number of studies correlating the Notch pathway with the development of the human ovaries is very low. Kristensen et al. presented transcriptional profiles of human preantral follicles and expression of Notch components that are dynamically regulated during follicle growth. Expression of Notch core members in human ovaries is slightly different in comparison with mammalian, although some common patterns may be recognized. In particular, JAG1, HES1, and HEY2 were upregulated in preantral follicles in contrast to NOTCH2, NOTCH3, NOTCH4, JAG2, HES4, HES5, and HES6, whose expression was lowered. Besides, HEY1 expression was dependent on the size of the preantral follicle (Kristensen et al., 2015). In turn, all Notch receptors (NOTCH1–4) and Jagged ligands (JAG1/2) were abundantly expressed in human cumulus granulosa cells (Tanriverdi et al., 2013).

The human endometrium is the tissue constantly being remodeled along with the menstrual cycle. Changes preceding ovulation involve the proliferative phase followed by a secretory phase of differentiation accompanied by morphological and functional alterations to become responsive in a limited time frame. Finally, the cycle continues to the late secretory phase and menstruation (Paiva et al., 2009). The Notch pathway is thought to regulate numerous biological processes including cell invasion, survival, apoptosis, and differentiation that are the essence of endometrial remodeling (Leong and Karsan, 2006). To date, very little is known about Notch signaling in the development of normal endometrium including endometrial stromal cell decidualization (Afshar et al., 2012). Some of the Notch members have been already identified in the endometrium throughout the menstrual cycle, although these findings were mainly dedicated to endometrial carcinoma and were inconclusive (Cobellis et al., 2008; Mitsuhashi et al., 2012). More recent investigations of Sinderen et al. localized Notch1 in both the endometrial glandular and luminal epithelium with the highest expression in the secretory phase, whereas Notch3 was detected in the endometrial luminal epithelium in the proliferative phase. Among ligands, Jag1 and Dll4 were found in the glandular and luminal epithelium with elevated levels in the secretory phase of the cycle, similarly to Dll1; however, the expression of the latter protein was restricted to the glandular epithelium only. Hes was moderately expressed in the glandular and luminal epithelium with elevated levels in the secretory phase; nevertheless, it was not clearly stated which particular Hes protein is mentioned (Van Sinderen et al., 2014).

Apart from the insufficiency of data directly involving Notch signaling in the development of normal endometrial tissue in humans, this pathway may be indirectly associated with its well-known functionality. More recent studies revealed that Notch participates in angiogenesis during uterine decidualization through *in vivo* studies in murine models, suggesting that the Notch pathway likely functions in mammalian decidual angiogenesis via coordinating VEGFR signaling in endothelial cells (Garcia-Pascual et al., 2014, p. 4; Shawber et al., 2015).

The female reproductive system is primarily formed from Müllerian ducts, which in turn give rise to, i.a., the oviducts, uterus, as well as cervix/endocervix and vagina, and is accomplished through the mesenchymal-to-epithelial transition (MET) and EMT. During embryogenesis, the Müllerian ducts are derived from the coelomic epithelium, initially originating from the intermediate mesoderm. Cells localized within the latter tissue undergo partial MET to form mesoepithelial cells lining the coelomic epithelium followed by either typical MET to form the epithelium of the Müllerian ducts or EMT to form the Müllerian ducts mesenchyme (Kobayashi and Behringer, 2003). Furthermore, the outer parts of Müllerian ducts fuse and form the urogenital canal, finally giving rise to the vagina, cervix, and uterus. The cervical lining undergoes a subsequent transition into the squamous type of epithelium, although despite the common origin of epithelium that is shared with a vagina, the phenotypic differences are thought to arise from other causes, i.e., mesenchymal signals driving the fate of epithelial cells during the squamous transformation of Müllerian vaginal epithelium that involves expression of p63, a transformation-related protein encoded by TP63 gene (Ince et al., 2002). Notably, canonical Notch signaling was recognized in the specification of mesodermal cells during early embryogenesis through regulation of key TFs such as GATA family, Snail, and Twist, which are commonly activated in mesoderm formation. Moreover, a significant contribution of Notch signaling was also reported in EMT through the upregulation of Snail that is in turn required for mesoderm formation (Grego-Bessa et al., 2004; Timmerman et al., 2004). Also, Ferguson et al., by employing Amhr2-cre transgenic murine model of conditionally active NOTCH1 in the mesenchyme of the developing Müllerian duct, oviduct, uterine stromal cells, and granulosa cells in the ovary, demonstrated multiple developmental abnormalities, thus emphasizing the great importance of proper Notch signaling in the development of female reproductive tract (Ferguson et al., 2012, 2016). Additionally, the Notch-p63 regulatory loop has been established during embryogenesis by Tadeu and Horsley (2013), presuming that the formation of the cervix is also driven by the Notch pathway.

The endometrium of primates is characterized by a high and unique capacity to self-regenerate that occurs through a coordinated sequence of events involving strict regulation of differentiation of uterine progenitors accompanied by the promotion of an immune environment favoring the process of wound healing (Gellersen and Brosens, 2014). As aforementioned, Notch signaling is involved in the maintenance of progenitor cells, and its unique signature was found within human endometrial progenitors (Gargett et al., 2012). Moreover, few recent studies reported abrogation of endometrial regeneration and re-epithelialization with further consequences through deregulation in RBPJ expression, hence exposing the role of the Notch pathway in the functioning of the normal endometrium (Zhang et al., 2014a; Strug et al., 2018).

### Notch in Cancer Development and Progression

To elucidate and broaden current insight into Notch roles as well as its contribution in the carcinogenesis of female tract organs such as breast, cervix, ovary, and uterine endometrium, we performed global analysis profiling expression of the Notch pathway at two levels of signaling: first, involving the core members, and second, involving downstream effectors targeted by *HES/HEY* genes that complement the literature review.

#### Methodology

Population structure and phenotype heterogeneity between major subtypes of BC, CC, OV, and EC accompanied by normal tissues were studied by applying the uniform manifold approximation and projection (UMAP) method, recently emerging as a novel machine learning approach for dimension reduction in large transcriptomic data, preceded by principal component analysis (PCA). To date, PCA was mainly applied as a first-line tool for the reduction of data dimensionality, especially in genomics. However, principal components (PCs) of the highest variance exhibit included information along with an increase in sample size at a very slow pace; thereby, multiple two-dimensional projections of lower variance are typically investigated to explore the data. In proceeding so, features of more subtle character may be tangled within projections. To bring such features to daylight in a two-dimensional system, non-linear transformation methods could be a more appropriate approach that emphasizes the local structure of the data. One of the commonly used non-linear methods is t-distributed stochastic neighbor embedding (t-SNE), although it struggles with datasets of large size, conversely to UMAP. By UMAP, a common practice is to initially reduce burden within data through applying PCA followed by reduction of dimensions projected to leading PCs and therefore extracting the only meaningful structure of given population while filtering out confounding noise (for those interested, principles of UMAP approach in the context of genomic data are very well described in Diaz-Papkovich et al., 2019). The spatial analysis was additionally enriched with mutations and CNV data as well as the clinical outcome of the core Notch members [i.e., disease-free survival (DFS) and overall survival (OS) analyses]. Arbitrarily made classification of patients into subgroups based on median gene expression, which is a common approach to include variables of continuous character (such as gene expression) in survival analyses, may result in misleading or insignificant conclusions due to improper stratification of patients. Therefore, the algorithm of cutpoint optimization accompanying DFS/OS analysis was employed. In brief, DFS/OS analysis is preceded by optimal cutpoint determination, which is defined as a cutpoint of the most significant split enabling patients to be categorized according to favorable or unfavorable prognosis based on the expression of a particular gene.

## Results and Discussion

**Figure 2:**
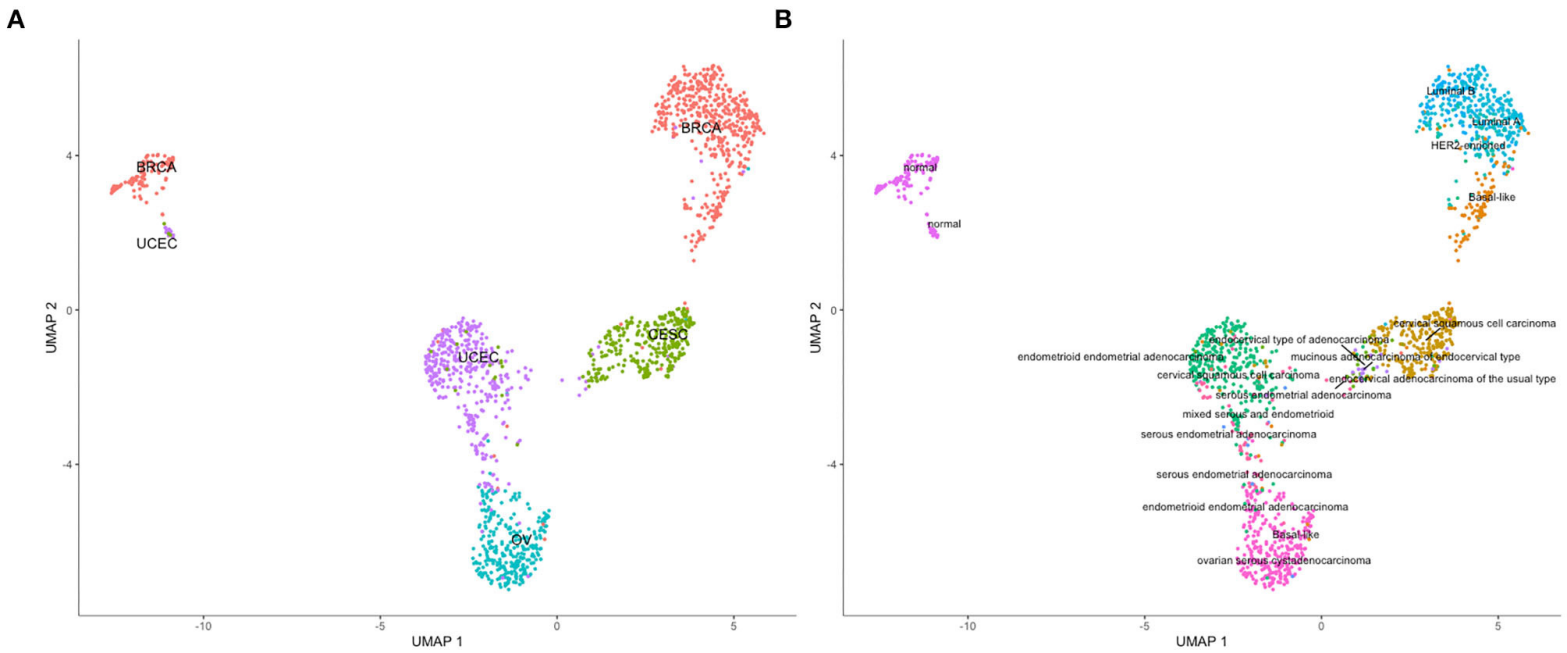
Spatial profiling of BC, CC, OV, and EC accompanied by normal tissues regarding the expression of the Notch core components. **(A)** shows the distribution of the cancer types accompanied by the normal tissues and the **(B)** specifies subtypes of the tumors with a separate cluster of basal-like BC and normal tissues, independently of origin.

**Figure 3:**
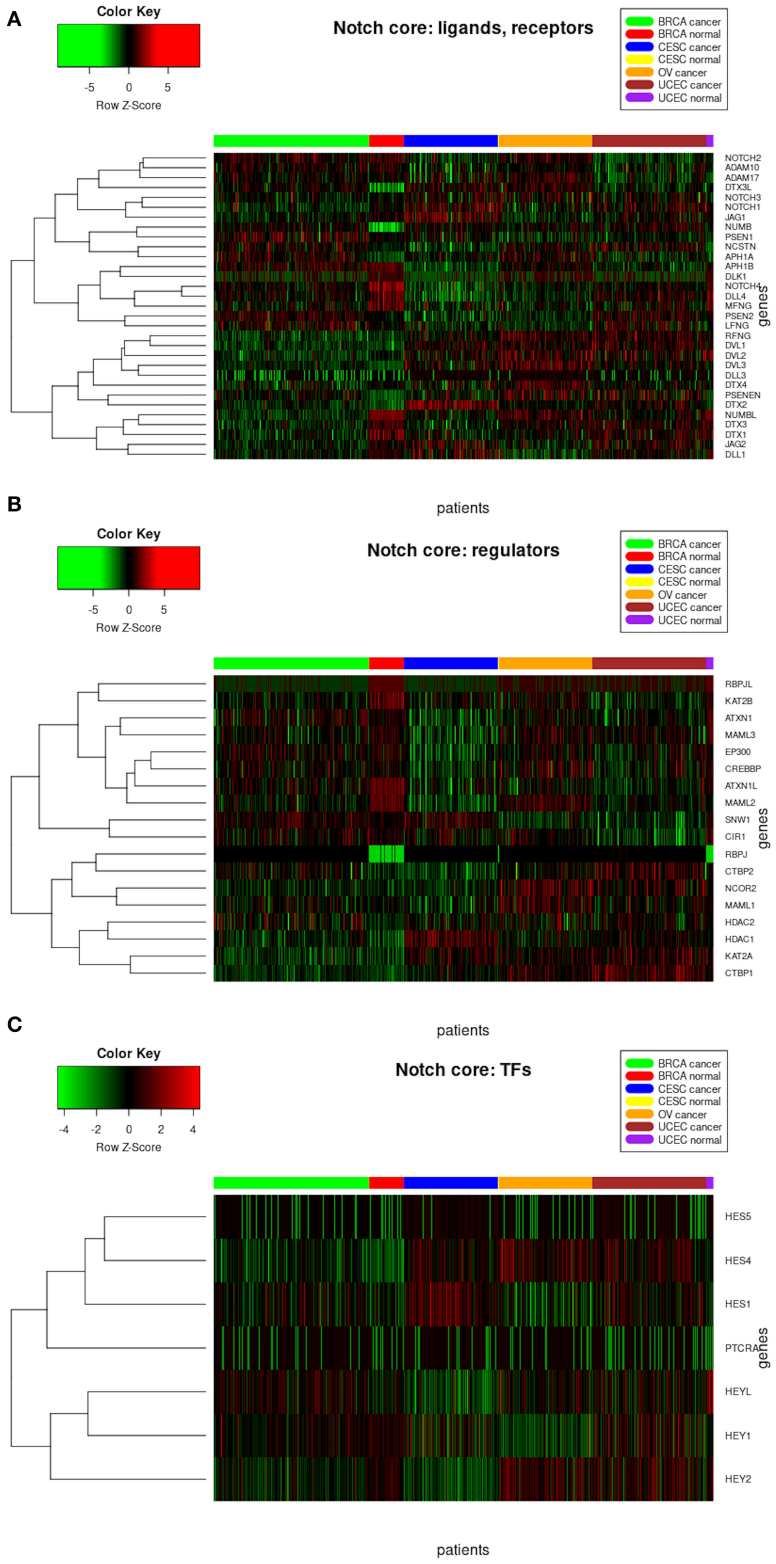
Heatmap reflecting differential gene expression of Notch core members in cancerous and normal tissues of breast, cervix, ovary, and uterine endometrium divided into functional groups of **(A)** receptors, ligands, and associated regulators; **(B)** modulators of signal; and **(C)** Notch-specific transcription factors.

The analysis of resultant total expression of 56 Notch core components among BC, CC, OV, and EC patients revealed clear spatial partitioning of each cancer type within UMAP spaces. Normal tissues of all organs have been clustered together, independently of tissue of origin, which suggests a nearly common profile of Notch signaling in normal sex hormone-dependent female tissues. If so, we addressed the question of how the Notch signaling alters in cancerous tissue such as BC, CC, OV, and EC vs. normal tissues. BC and CC tended to be the most distinct tumors regarding Notch core, as they formed separate clusters of samples, well-differentiated from each other and simultaneously from OV and EC in UMAP1 and UMAP2, respectively. OV and EC in turn seemed to be more similar to each other regarding UMAP1, albeit still different from BC and CC in UMAP2. Moreover, profiles of Notch core reflected internal partitioning of BC samples referring to PAM50-based classification, with basal-like subtype manifesting characteristics of a separate cluster of samples (Figure 2). The profiles of the Notch core components expression are shown in Figure 3.

### Signaling by the Core—Ligands, Receptors, and Modulators

To date, the role of Notch and its core members has been of great research interest in various tumors. Starting with BC, the investigations conducted by Stylianou et al. became iconic in the field presenting the aberrant expression of Notch ligands, receptors as well as target genes among different BC cell lines. It was shown that attenuation of Notch signaling could revert the transformed phenotype of human BC *in vitro* (Stylianou et al., 2006). In particular, the available literature presents Notch1 as an oncogene. Its overexpression has been repeatedly correlated with BC progression as well as worse OS and DFS (Ercan et al., 2011; Yuan et al., 2015) and contributed to development and transition from DCIS to the invasive form of cancer (Farnie et al., 2007; Yuan et al., 2015). Additionally, Notch1 is involved in metastasis as high expression of NICD1 was attributed with sentinel lymph-node positive patients (Wieland et al., 2017). These findings were confirmed in a large bioinformatic meta-analysis involving 4,000 cases of human BCs correlating Notch signaling with increased risk of disease recurrence (Abravanel et al., 2015). However, alterations of Notch1 were reported predominantly in ER+/PR+/HER2+/– BCs (Dai et al., 2015), whereas mutations were more prevalent in HER2-negative tumors (Yi et al., 2017). As shown by numerous research, the role of Notch signaling in metastasis is even more eminent due to contribution in the process of EMT. Leong et al. showed the dependency of Jag1-Notch1-SLUG related to E-cadherin signaling. In particular, activation of Notch1 led to SLUG-facilitated repression of E-cadherin (Leong et al., 2007). Jag1-mediated signaling by Notch increased expression of mesenchymal markers such as N-cadherin, vimentin, Slug, Snail, Zeb1, as well as β-catenin to the detriment of E-cadherin repression (Chen et al., 2010; Brabletz et al., 2011; Bolos et al., 2013; Jian et al., 2013; Liu et al., 2014). Notch activity was also reported in hypoxia-induced EMT. In-depth studies revealed the involvement of Notch1 in hypoxia and CSC-related metastasis (Xing et al., 2011) and, hand in hand with high HIF, predicted worse patient outcomes and thus contributed to more aggressive BC phenotype (Ercan et al., 2012). Finally, Notch1 and Jag1 were related to tumor dormancy in the bone marrow environment able to induce metastasis through the Notch1/STAT3/LIFR signaling axis (Johnson et al., 2016), though overexpression of JAG1 was sufficient to induce bone metastasis (Sethi et al., 2011). Other studies reported Jag1 promoting angiogenesis in neighboring endothelial cells (Reedijk et al., 2005). Additionally, the significance of Jag1 was reported mainly in TNBC exhibiting high levels of NF-kB signaling. The induction of Jag1 in a NF-kB-dependent manner led to the expansion of CSC populations; however, it was observable only among basal-like subtypes (Yamamoto et al., 2013). Moreover, Boareto et al. in a series of their articles depicted the asymmetry between Notch signaling through Delta and that through Jagged affecting the phenotype acquired by the cell implicating worse clinical outcome of the disease. As a brief recap, Notch-Delta signaling allows only two states: Sender or Receiver; however, due to the Delta-Jagged asymmetry, the third possible state of a hybrid Sender/Receiver has arisen, whose relevance was revealed in angiogenesis and EMT (Boareto et al., 2015b). During angiogenesis, the endothelial cells adopt one of the phenotypes: a tip, leading to the formation of branching vessels, and a stalk, proliferating to develop the vessel. Hereby, Boareto et al. demonstrated the diversified effects of Delta-Jagged asymmetry in selecting the tip cell in response to VEGF, an angiogenic growth factor. Specifically, the domination of Notch-Jagged over Notch-Delta signaling destabilizes the tip and stalk cell fates toward the hybrid tip/stalk phenotype, leading to the chaotic, poorly perfused angiogenesis due to the formation of a new sprout that can migrate and develop filopodia. Thus, a hybrid tip/stalk phenotype gives the leading cell an advantage to rapidly exchange its position with a neighbor stalk to induce fast vessel branching that ensures an efficient supply of oxygen to rapidly growing tumors (Boareto et al., 2015a) and might be an explanation why Jag1 overexpression is favored in the tumor environment, especially in tumor–stroma cross-talk (Li, 2014), while Dll4 acts as a brake on sprouting angiogenesis and supports physiological angiogenesis (Suchting et al., 2007). On the other hand, it is also believed that the Sender/Receiver hybrid state occurs in cells that underwent partial EMT and are progressing, hence enabling such cells to maintain the meta-stable hybrid epithelial/mesenchymal (E/M) phenotype. The Notch-Jagged signaling has been therefore a hallmark of more aggressive tumor characteristics linked with metastasis and tumor relapse through promoting the E/M hybrid and CSC-like characteristics. Bocci et al. showed that Notch-Jagged signaling might facilitate the formation of hybrid E/M cell clusters potentiating to dislodge from the primary tumor as clusters of CTCs aggravating tumor progression via tumor–stroma interactions (Bocci et al., 2019). These findings confirmed earlier research demonstrating Jag1 among metastasis effectors promoting the remodeling of metastasis niche (Cheung et al., 2016). Finally, *JAG1* knockdown significantly reduced tumor emboli formation in SUM149 BC cells (Bocci et al., 2019).

In the present study, we aimed to include the effects of Notch core alterations evaluated at two different levels: (1) comparison between cancerous vs. normal tissue, and (2) determination of expression cutpoint splitting patients into two subgroups of more/less favorable clinical outcome referring to the relative level of expression (above/below the estimated cutpoint) within cancer only marking the oncogenic or suppressive character of a particular gene. In the TCGA data, we observed opposed trends in NOTCH1 expression of ~2-fold decrease among BC patients in comparison with normal breast tissue (Table 2). Nonetheless, OS and DFS analyses revealed decreased NOTCH1 expression associated with a more favorable prognosis (HR = 1.66, *p* = 0.047; cutp: HR = 3.14, *p* = 0.006; maxstat: HR = 3.13, *p* = 0.006, respectively). Since the lowered range of expression within BC cases was more favorable in terms of BC prognosis, this finding reaffirmed the oncogenic character of *NOTCH1* during breast carcinogenesis (**Tables 4**, **5**). Similarly, JAG1 was doubly decreased in BC vs. normal tissue (Table 2), although the lowered expression within BC only was more favorable regarding DFS, it confirmed the involvement of *JAG1* in the mechanism of the recurrence (cutp: HR > 100, *p* = 0.043; **Table 5**).

**Table 2:** Summary statistics on Notch core components including logFC accompanied by frequency of mutations and CNVs.

	**BRCA**			**CESC**			**OV**†		**UCEC**		
	logFC*	**mut [%]**	**CNV [%]**	logFC*	**mut [%]**	**CNV [%]**	**mut [%]**	**CNV [%]**	logFC*	**mut [%]**	**CNV [%]**
*ADAM10*	−0.17	–	–	0.74	–	–	–	–	−0.02	–	–
*ADAM17*	−0.21	–	–	1.65	–	–	–	–	0.52	–	–
*APH1A*	0.79	–	12.3	0.24	1	3.1	0.3	10.2	0.52	–	7.1
*APH1B*	−0.63	–	–	−1.79	–	–	–	–	−1.4	–	–
*ATXN1*	−0.047	–	–	−1.05	–	–	–	–	−1.21	–	–
*ATXN1L*	−0.93	–	–	−0.36	–	–	–	–	−0.61	–	–
*CIR1*	−0.14	–	–	−0.67	–	–	–	–	−1.06	–	–
*CREBBP*	−0.1	1.8	4.9	−0.35	7.2	1.7	2.2	4.2	−0.47	8.9	0.9
*CTBP1*	0.19	–	–	−0.15	–	–	–	–	0.32	–	–
*CTBP2*	0.4	–	–	−0.3	–	–	–	–	0.27	–	–
*DLK1*	−7.38	–	–	–	–	–	–	–	–	–	–
*DLL1*	−1.37	–	–	−0.7	–	–	–	–	−1.76	–	–
*DLL3*	1.13	–	–	–	–	–	–	–	1.21	–	–
*DLL4*	−0.82	–	–	−2.5	–	–	–	–	−1.24	–	–
*DTX1*	−2.31	0.6	0.2	−0.09	0.5	0.3	0.3	2.2	−0.25	2.8	1.1
*DTX2*	0.56	–	–	1.76	–	–	–	–	1.01	–	–
*DTX3L*	2.12	–	–	1.03	–	–	–	–	0.01	–	–
*DTX3*	−1.44	–	–	−0.94	–	–	–	–	−0.22	–	–
*DTX4*	−0.89	–	–	0.83	–	–	–	–	0.04	–	–
*DVL1*	0.23	–	–	0.07	–	–	–	–	0.18	–	–
*DVL2*	−0.24	–	–	−0.74	–	–	–	–	−0.53	–	–
*DVL3*	0.38	–	–	0.74	–	–	–	–	0.4	–	–
*EP300*	−0.23	1.6	0.2	−0.16	10.8	2.4	0.3	2.5	0.02	8.9	1.7
*HDAC1*	0.36	0.8	0.8	1.07	–	0.7	0.3	6.5	0.23	1.6	2.6
*HDAC2*	0.38	–	–	0.54	–	–	–	–	0.14	–	–
*HES1*	−0.17	–	–	1.39	–	–	–	–	0.67	–	–
*HES4*	1.04	–	–	1.2	–	–	–	–	1.54	–	–
*HES5*	−0.28	–	–	1.26	–	–	–	–	0.11	–	–
*HEY1*	−0.98	0.2	9.8	−0.3	0.5	1	–	8.6	0.6	–	2.8
*HEY2*	−2	–	–	−1.51	–	–	–	–	−0.02	–	–
*HEYL*	0.85	–	–	−3.38	–	–	–	–	−2.15	–	–
*JAG1*	−0.74	–	–	0.38	–	–	–	–	−0.07	–	–
*JAG2*	−0.95	–	–	0.66	–	–	–	–	0.67	–	–
*KAT2A*	0.04	–	–	0.72	–	–	–	–	0.25	–	–
*KAT2B*	−1.4	–	–	−1.1	–	–	–	–	−1.23	–	–
*LFNG*	0.38	–	–	−0.04	–	–	–	–	0.43	–	–
*MAML1*	−0.01	–	–	−0.09	–	–	–	–	−0.19	–	–
*MAML2*	−2.54	1.2	2	−0.5	2.6	4.4	0.9	8.1	−0.11	3.2	1.5
*MAML3*	−0.55	–	–	−1.13	–	–	–	–	−1.14	–	–
*MFNG*	−1.48	–	–	−2.38	–	–	–	–	−1.31	–	–
*NCOR2*	0.03	1	1.8	−0.2	4.1	–	0.3	3.5	0.2	6	2
*NCSTN*	0.43	0.2	10.7	0.55	1	3.1	–	5	0.94	3.2	4.6
*NOTCH1*	−0.81	0.6	1.2	0.83	5.7	1	1.3	4.5	0.03	3.2	2.4
*NOTCH2*	−0.46	2	12.1	−0.18	3.6	2.7	1.3	11	−0.38	5.6	6.1
*NOTCH3*	−0.03	1	2	0.82	4.1	2.1	0.9	16.6	0.55	6.5	7.2
*NOTCH4*	−1.67	1	1	−2.46	6	2.4	1.6	6.4	−1.22	4.8	1.9
*NUMB*	−2.37	–	–	−1.74	–	–	–	–	−1.42	–	–
*NUMBL*	1.84	–	–	0.03	–	–	–	–	0.35	–	–
*PSEN1*	0.22	–	–	0.37	–	–	–	–	0.21	–	–
*PSEN2*	0.71	–	–	−0.85	–	–	–	–	0.09	–	–
*PSENEN*	1.02	–	–	0.68	–	–	–	–	1	–	–
*PTCRA*	1.42	–	–	0.61	–	–	–	–	2.09	–	–
*RBPJL*	–	–	–	–	–	–	–	–	−9.63	–	–
*RBPJ*	–	–	–	–	–	–	–	–	–	–	–
*RFNG*	0.01	–	–	−0.65	–	–	–	–	0.19	–	–
*SNW1*	0.06	–	–	0.05	–	–	–	–	−0.66	–	–
	**BRCA**			**CESC**			**OV**		**UCEC**		
	**mut [%]**		**CNV [%]**	**mut [%]**		**CNV [%]**	**mut [%]**		**CNV [%]**	**mut [%]**	**CNV [%]**
*TP53*	32.9		1.8	4.6		0.3	87.7	1.7	27.8		1.1
*DNMT1*	0.6		1.6	1.5		2.7	0.6	11.2	3.6		5.8
*HDAC1*	0.8		0.8	–		0.7	0.3	6.5	1.6		2.6
*HDAC4*	0.6		1	1		5	0.6	3.4	4.8		1.3
*HDAC7*	–		0.6	0.5		0.7	0.3	2.6	2.8		3.2

† *No comparison of expression due to lack of normal tissue samples in case of OV*.

* *In cancerous vs. normal tissue*.

Similar findings were reported in OV, where Notch1 was associated with cellular growth through increased proliferation rate and colony formation capacity by NICD1 (Hopfer et al., 2005), similarly as observed among various OV cell lines (OVCAR3, SKOV3, CaOV3) (Rose et al., 2010). Immunohistochemistry (IHC) revealed the presence of Notch1 in 95% of serous OV; however, it was additionally observed in the marginal percentage of matched benign and normal ovarian controls (8 and 6%, respectively) (Wang et al., 2010), as opposed to NICD1, which was undetectable (Kluk et al., 2013). Few other studies associated Notch with OV recurrence, a phenomenon currently attributed to a tumorigenic and therapy-resistant subpopulation of TICs/CSCs (O'Connor et al., 2014). Consistent with the role of Notch in the maintenance of SCs, increased expression of NOTCH1 was found among spheroid ovarian CSCs (Zhang S. et al., 2008). Additionally, NOTCH1 significantly differentiated progression-free survival (PFS) according to TP53 mutation status, and its overexpression correlated with worsened prognosis, although no association with OS was observed (Zhou et al., 2016). In the case of EC, Notch1 showed higher expression compared to normal endometrium, independently of layer or phase of the menstrual cycle, as shown by IHC. The expression increased with the advanced International Federation of Gynecologists and Obstetricians (FIGO) stage. It was also associated with deep myometrial invasion, vessel involvement, and ovarian metastasis and translated into the poorer OS, especially in combination with JAG1 (Mitsuhashi et al., 2012). Cobellis et al. identified Notch1 upregulation in hyperplasia and carcinomas compared to polyps, concluding the oncogenic role in EC tumorigenesis (Cobellis et al., 2008). Importantly, the evaluation of NOTCH1 expression at the RNA level showed an inverse suppressive character of the receptor, in contradiction to the previous studies (Jonusiene et al., 2013). Our analysis showed that no significant alterations of NOTCH1 were found among EC patients, although survival analysis revealed the dualistic effects. Lowered NOTCH1 correlated with improved survival (cutp: HR = 2.22, *p* = 0.026; maxstat: HR = 3.56, *p* = 0.002; **Table 4**), whereas its elevation was associated with favorable DFS prognosis (cutp: HR = 0.376, *p* = 0.007; maxstat: HR = 0.36, *p* = 0.022; **Table 5**).

Regarding CC, Notch has been revealed as a key mechanism in transformation and cancer progression. The primary oncogenic mechanism involved activation of NICD1 that was shown to phenocopy activation of Ras (considered as second hit accompanying HPV-related E6/E7 oncogenic activity in transforming immortalized HaCaT keratinocytes) (Rangarajan et al., 2001, p. 1). Tumorigenic properties of CC cells are modulated by Notch1 and RhoC. Co-expression of both molecules was observed in primary CC biospecimens and Notch1 KO resulted in the downregulation of RhoC followed by a decrease in cell migration and invasion *in vitro* (Srivastava et al., 2010). Nevertheless, some studies also showed the tumor-suppressive character of Notch signaling in CC. For instance, high expression of Notch1 resulted in growth arrest of cervical tumor-derived cells (Talora et al., 2002, 2005; Wang et al., 2007). Greater levels of Notch components were observed in CC samples compared to normal tissues or high-grade lesions (Daniel et al., 1997; Campos-Parra et al., 2016; Rong et al., 2017). Besides, most invasive CCs exhibited Notch1 expression, prevalently observed within the cytoplasm, conversely to cervical intraepithelial neoplasia (CIN) samples, where nuclear localization of Notch1 was identified. The former finding was also correlated with CC progression, although the latter indicated poorer clinical outcomes (Vazquez-Ulloa et al., 2018). Tripathi et al. reported in turn that both nuclear and cytoplasmic Notch1 expression was decreased through the progression of cervical lesions, from non-neoplastic to precancerous lesions to a tumor, and this finding was additionally confirmed (Tripathi et al., 2014). We found doubly higher expression of NOTCH1 in cancerous tissue vs. normal tissue (Table 2) and lowered expression that predicted better survival of CC patients (cutp: HR = 1.73, *p* = 0.058; maxstat: HR = 1.67, *p* = 0.049; **Table 4**).

To date, very little is known about Notch2, especially in the context of the remaining receptors that have been widely described; for instance, its relevance in tumorigenesis of EC or CC has not been established and remains elusive. What has been established is a regulatory role of a transcriptional and functional character that Notch2 plays in governing signals from Notch1 and Notch3 in BC (Shimizu et al., 2002). Also, its alterations affected the luminal cellular hierarchy during the specification of mammary epithelial lineages (Sale et al., 2013). Among BC cases, Notch2 mutations were the most prevalent (Lee et al., 2016) and correlated with better prognosis, especially visible in low-grade tumors (Kim et al., 2016). Our study confirmed the increased CNV frequency of 12.1% in NOTCH2 with no relevance to the level of expression or survival in BC (Tables 2, **4**, 5). In OV, higher expression of NOTCH2 correlated with worse PFS, independently of TP53 mutations, especially in grade II (Parr et al., 2004). We observed similar associations of NOTCH2 with OS and DFS as Zhou et al. with PFS. In particular, lower expression was correlated with better prognosis, regardless of the status of TP53 mutations (**Tables 4**, **5**). Our study also revealed an interesting finding that NOTCH2 significantly modulates the survival of EC patients. The initial evaluation of cutpoints stratifying EC patients into subgroups of differential survival turned our attention to the diverse results computed by the algorithms (cutp: cutpoint = 3285, HR = 2.42, *p* = 0.015; maxstat: cutpoint = 1299, HR = 7.31, *p* = 0.02; **Tables 4**, **5**). We thereby assumed that there is a potential third distinguishable group of patients of moderate survival prognosis regarding NOTCH2 and confirmed that with a proper algorithm, finally showing the improving survival prognosis with decreasing NOTCH2 expression (**Table 6**).

Notch3 tends to exert dualistic, i.e., oncogenic and suppressive roles. On one side, Notch3 has been widely shown in mammary carcinogenesis (Dievart et al., 1999; Yamaguchi et al., 2008; Pradeep et al., 2012; Yi et al., 2017), specifically increasing proliferation of luminal cells through cyclin D1, Myc, and Akt (Pradeep et al., 2012). This finding refers to the earlier established regulatory role of Notch3 in the commitment of luminal cells lineage from bipotent progenitors (Raouf et al., 2008). Long-term hormonal therapies were frequently shown as eventually leading to therapy resistance, independently of ER status. Indeed, Notch3 has been indicated as a factor that may contribute to the acquisition of the resistance through the IL6/STAT3/Notch3 axis that causes a departure from metabolic dormancy. Inhibition of IL6 in BC *in vitro* resulted in the downregulation of Notch3 followed by resensitization to hormonal therapies, e.g., tamoxifen (Sansone et al., 2016). It was also demonstrated that in response to TGF-β produced by bone marrow osteoblasts, increase in Notch3 and Jag1 promoted osteoblast differentiation and bone metastasis formation (Zhang et al., 2010; Sethi et al., 2011). In contrast, overexpression of Notch3 could also be tumor suppressive, as shown by Chen et al., through the upregulation of Cdh1 leading to the accumulation of p27^Kip1^ and cell cycle arrest at the G0 to G1 phase transition *in vitro* (Chen et al., 2016, 1). On the other side, Notch3 may inhibit EMT in BC through a novel mechanism comprising the upregulation of GATA3 (Lin et al., 2018, 3). Notch3 was also negatively correlated with chemoresistance (Gu et al., 2016, p. 3). Concerning Notch4, its oncogenic role has been to date mostly described in murine models (reviewed in detail elsewhere; Politi et al., 2004). However, Notch4 was also related to the formation and maintenance of CSCs in BC that surpassed Notch1 in efficacy in that context (Azzam et al., 2013). On the other hand, Notch4 was currently presented to sensitize BC cells *in vitro* to TRAIL-induced apoptosis (Naik et al., 2015). It was also correlated with poor prognosis followed by anti-estrogen treatment, although short-term therapies resulted in increased activity of CTCs through Jag1/N and otch4 activation (Simoes et al., 2015). Our analysis indicated the tumorigenic character of NOTCH4, especially associated with disease recurrence (cutp: HR = 5.37, *p* = 0.002; maxstat: HR = 5.24, *p* = 0.002; **Table 5**) and survival (HR = 1.96, *p* = 0.053; **Table 4**) in BC; nonetheless, the expression dropped as compared to the normal tissue (logFC = −1.67; Table 2).

The Notch signature in OV was primarily recognized in studies aiming to identify diagnostic markers of epithelial OV in human samples and *in vitro* cultures. Interestingly, Notch3 overexpression has been accompanied by amplification localized within NOTCH3 locus that has been identified among serous high-grade OV [confirmed by multiple techniques: SNP genotyping, fluorescent *in situ* hybridization (FISH), IHC] and studies including TCGA Network (Park et al., 2006; Nakayama et al., 2007). Our study confirmed the former of the increased prevalence of CNVs within NOTCH3 (16.6%) in OV (Table 2). Among ligands, Jag1 was mainly identified within OV cells and surrounding peritoneal mesothelial cells. Additionally, it was shown that Jag1 activation of signaling through Notch3 promoted cell proliferation and adhesion (Choi et al., 2008). Jung and collaborators conducted expression profiling of Notch in serous OV vs. benign tissues and reported elevation of NOTCH3, JAG1, and JAG2 as well as corresponding higher levels of Notch3 and Jag1 proteins. Furthermore, NOTCH3 was correlated with poor OS and resistance to chemotherapy, although at the protein level, Notch3 was correlated with the advanced stage of the disease, lymph node, and distant metastasis (Jung et al., 2010). High NOTCH3 was, in contrast, more favorable regarding PFS (Zhou et al., 2016). Our analysis did not confirm the relevance of NOTCH3 in either OS or DFS, although we identified the beneficial effects of relatively higher JAG1 (HR = 0.612, *p* = 0.034) and JAG2 (HR = 0.675, *p* = 0.042) in the latter (**Tables 4**, **5**) during ovarian carcinogenesis.

Another study involving NICD3 *in vitro* cultures with lowered endogenous Notch concentration demonstrated elevated expression of SC-associated genes such as NANOG and OCT4, thereby highlighting the relevance of Notch signaling in CSC biology (Park et al., 2010). Notch was also implicated in promoting tumor invasiveness and metastasis through participation in the process of EMT, which in OV has been associated with chemoresistance and SC-like characteristics (Marchini et al., 2013). It was shown that the upregulation of NICD3 in the serous OV cell line (OVCA429) triggers EMT. This finding was confirmed by noticeable alterations in cellular morphology conformed to remind fibroblasts and differential levels of mesenchymal markers and epithelial markers (high Slug, Snail, α-actin vs. low E-cadherin). Moreover, the cells were resistant to carboplatin-induced apoptosis in comparison with control OVCA429 cells (without NICD3 overexpression) (Gupta et al., 2013). Moreover, several studies described the role of Notch signaling in angiogenesis, specifically in serous OV. Microarray-based differential gene expression (DGE) analysis comparing profiles between endothelial cells from high-grade serous OV and endothelial cells from benign ovaries revealed upregulation of JAG1, whose subsequent RNAi silencing reduced tube formation and migration of endothelial cells (Lu et al., 2007). The IHC-based analysis reflected in turn overexpression of Dll4 in tumor and endothelium in over 70% of OV samples that were ultimately correlated with worse OS in contrast to Dll4-low samples (Hu et al., 2011). Conversely, we found beneficial effects of DLL4 upregulation in the context of patients' survival (cutp: HR = 0.71, *p* = 0.02; maxstat: HR = 0.725, *p* = 0.04; **Table 4**) and OV recurrence (HR = 0.661, *p* = 0.04; **Table 5**); admittedly, the evaluation of the trend was based on the level of mRNA.

The case of resistance to platinum-based therapies that is frequently observed in OV and results in recurrence of the disease is still being widely discussed. It also remains a major obstacle, whose overcoming is of the greatest urgency concerning patients' prognosis. Regarding well-established Notch association with CSCs and further relevance of CSCs in a mechanism of acquiring drug resistance, the Notch pathway has recently become a major focus in attempts to understand failures of OV management. Generally, drug-resistant and self-renewing CSCs have been considered as a potential cause of disease recurrence among advanced stage OV patients post platinum-based therapy that is co-observed with multidrug-resistant (MDR) phenotype. Currently applied therapies target the bulk of tumor cells, which rapidly divide and do not exhibit CSC-related characteristics; therefore, the reduction in primary tumor mass is observed, although it simultaneously extricates the niche of drug-resistant CSCs (Gupta et al., 2009). Recent studies reported the specific involvement of Notch3 and Jag1 (Munoz-Galvan et al., 2019) in the mechanism of OV resistance to treatment and its recurrence. In the former, upregulation of NOTCH3 was observed in tumor high-grade serous OV vs. normal ovarian samples and correlated with significantly shorter survival. Moreover, the cluster of Notch signaling was identified in the network-based analysis and related to the prediction of OV response to platinum treatment. These findings were confirmed *in vitro* involving tumorspheres enriched in CSCs, showing elevated Notch signaling, especially NOTCH3; similar observation was made among particular OV patients resistant to platinum therapy. Finally, inhibition of Notch signaling via GSI *in vitro* implicated in a significant reduction in the formation of tumorspheres treated with either cis- or carboplatinum (Munoz-Galvan et al., 2019). The latter investigations emphasized that, as was previously mentioned, the formation of CSCs is essentially regulated by EMT. In turn, the Notch pathway has been widely demonstrated as a critical regulatory mechanism of the EMT process as was also confirmed therein. Specifically, Jag1 seemed to have a more prevailing role in mediating EMT in cisplatin-resistant cells than Jag2, which agrees with earlier studies (Choi et al., 2008; Steg et al., 2011) defining Jag1 as the main ligand of the Notch pathway in OV. Furthermore, these investigations confirmed (in fact, already established; Androutsellis-Theotokis et al., 2006) the existence of cross-talk between Jag1 and STAT3 (major player of JAK/STAT signaling that determines cell polarity, especially in the progression of EMT in cancer), their physical interactions, and the effects of their deadly cross-talk leading to the promotion of the EMT and thus reinforced the invasion and migration capacity of cisplatin-resistant ovarian cancer cells both *in vitro* and *in vivo* (Yang et al., 2019, 3). Liu et al. as well-referred to Jag1-induced stemness and chemoresistance to platinum-based therapies in OV and surprisingly revealed GATA1 as upstream TF that targets Jag1, thereby activating the Notch pathway and ultimately resulting in OV progression and development of chemoresistance (Liu et al., 2020, p. 1). Therefore, Jag1 may be considered as a linking molecule between other signaling pathways that exert progression of OV in cooperation with Notch signaling. In addition to the aforementioned findings, it was shown that in the absence of the Wnt signaling, the Jag1-activated Notch pathway sustains the proliferation and migration of OV cells *in vitro* and mouse xenograft models (Bocchicchio et al., 2019).

The Notch pathway was also attributed to the dissemination of OV cells through evading cell death in a very specific manner. Generally, high-grade serous OV originates from fallopian tube ECM-exfoliated cells that underwent a tumorigenic transformation; thereby, it may be concluded that escape from anoikis and survival in an anchorage-independent manner is the essence of OV spread. Importantly, Notch has already been implicated in anchorage-independent survival, e.g., NOTCH3 drives resistance to anoikis (Brown et al., 2015, 2). A very recent study cross-referencing functional CRISPR/Cas9 genome-wide knockout screen as well as metabolomics and transcriptomics to identify pathways promoting a state of anchorage independency in high-grade serous OV demonstrated Notch enrichment (as the whole pathway) as well as specific alterations of NOTCH1 and NOTCH3. Moreover, since the Notch pathway contributes to fatty acid (FA) transport (Jabs et al., 2018) and angiogenesis, the conclusion was drawn that it promotes the spread of OV cells in part through FA metabolism, which makes the peritoneal omentum the site of first choice of OV cells to disseminate (Wheeler et al., 2019).

Finally, Notch receptors were correlated with the prognosis of OV patients. High NOTCH3 was more favorable regarding PFS in all OV cases. Elevated expression of NOTCH4 was in turn significantly correlated with more favorable OS in all OV cases; however, the difference in prognosis was not so evident; nonetheless, we also observed such correlation in our research (HR = 0.602, *p* = 0.04; **Table 4**). Further correlations with clinicopathological parameters were additionally established such as better OS prognosis regarding upregulated NOTCH4 among grade III OV patients (Zhou et al., 2016).

Significantly higher expression of Notch3, Jag1, and Dll4 was also reported among EC compared to normal endometrium, regardless of the layer of the endometrium (Mitsuhashi et al., 2012). Cobellis et al. examined in a similar way the expression of Notch4 and Jag1 in normal endometrial samples of pre- and postmenopausal women and compared it with unmatched pathologic samples including, i.a., EC. Conversely, Notch4, and Jag1 decreased with increasing histological grade. Concerning the above, the authors concluded that Notch4 exhibited a more suppressive character (Cobellis et al., 2008). DidŽiapetriene et al. reported alterations in Notch signaling evaluated at the RNA level (qPCR). The study included quantification of Notch receptors (NOTCH1–4), ligands (JAG1, JAG2, and DLL1), and HES1, and in turn revealed significant decrease in expression of all analyzed genes in EC compared to matched, adjacent non-tumor endometrium (Jonusiene et al., 2013; Lachej et al., 2019). Moreover, NOTCH4 and DLL1 were downregulated more likely in stage IB than IA tumors (Sasnauskiene et al., 2014). Our analysis revealed diverse alterations in the expression of Notch ligands and receptors. Conversely to NOTCH3, JAG2, and DLL3, NOTCH4, DLL1, and DLL4 showed lowered expression in EC in comparison with normal endometrium (Table 2). Consistent with the above, we also identified oncogenic effects of DLL3 on survival (cutp: HR = 2.29, *p* = 0.02; maxstat: HR = 2.6, *p* = 0.007; **Table 4**) and disease recurrence (cutp: HR = 3.15, *p* = 0.005; maxstat: HR = 2.95, *p* = 0.002; **Table 5**). Among ligands, JAG2 demonstrated similar effects on OS (cutp: HR = 2.17, *p* = 0.05; maxstat: HR = 2.16, *p* = 0.05; **Table 4**), whereas lowered expression strongly correlated with more favorable DFS (cutp: HR = 0.193, *p* < 0.001; maxstat: HR = 0.215, *p* < 0.001) the same as DLL1 (cutp: HR = 0.396, *p* = 0.009; maxstat: HR = 0.388, *p* = 0.012; **Table 5**). Regarding receptors, lowered expression of NOTCH3 was associated with better survival (HR = 2.6, *p* = 0.005; **Table 4**) and DFS prognosis (HR = 2.71, *p* = 0.006; **Table 5**), whereas NOTCH4 was insignificant.

To date, no reports on the significance of either Notch ligands or receptors (excluding Notch1) were found in the area of CC. Our study revealed a decrease in DLL1 and DLL4 expression in CC vs. normal tissue, conversely to JAG1 and JAG2, which were elevated (Table 2). Regarding the receptors, there is only one study from 2016 of Sun et al. that demonstrated overexpression of intracellular domains of Notch receptors (NICD1–4) significantly reducing cell proliferation in HeLa cells (Sun et al., 2016, p. 2). As we described earlier, the initiation of Notch cascade may occur in a non-canonical way, independently of receptor activation, and this fact affects the possibility of referring these findings to the classical evaluation of the receptor relevance. In our study, NOTCH3 showed ~2-fold lowered expression than in normal tissue, in contrast to NOTCH4, which was significantly elevated (Table 2). In the context of survival, ligands including DLK1, DLL1, DLL3, DLL4, and JAG1 as well as the NOTCH4 receptor were oncogenic, as a lowered expression of these molecules correlated with improved OS in CC, although with diverse effects on DFS. In particular, DLL3, JAG1, and NOTCH4 retained their relevance as in OS, whereas lowered DLL4 correlated with better prognosis (**Tables 4**, **5**).

Additionally, it is worth recalling the specific BC subtype, i.e., TNBC (esp. basal-like BC) characterized by very poor overall outcome increasingly regarded as a separate clinical entity. Recently, Notch signaling also emerged in pathogenesis and disease progression of TNBC. Its receptors have been related to multiple mechanisms reflecting the aggressive character of TNBC that were already described in previous sections, but deserves additional emphasis. Notch importance in TNBC starts from regulatory effects of Notch receptors on TICs behavior through the association of Notch signaling in maintenance and expansion of mammary CSCs and finally ending with a correlation between expression of Notch receptors and aggressive clinical course of the disease, including invasiveness and chemoresistance. Pathological activation of Notch1 has been considered as a key event in the etiology of TNBCs. Moreover, it contributed to a more aggressive phenotype of TNBC as well as the further progression of the disease, especially through Notch–EMT interactions (Giuli et al., 2019). The role of Notch2 has not been clearly explained in the context of TNBC, although *in vitro* studies suggested its ambiguous role in the pathogenesis. Signaling through Notch2 initiated by Jag1 and/or Dll4 together with FYN/STAT5 was reported to maintain the mesenchymal phenotype of cells in basal-like BC. It was demonstrated that Notch2 silencing via siRNA reduced expression of mesenchymal markers such as vimentin, Snai1/2, Twist, and Zeb1 (Lee et al., 2018). In turn, Notch3 was defined as an essential factor for TNBC to acquire more aggressive features. Lastly, elevated Notch4 promotes the mesenchymal phenotype and maintains stemness during the progression of TNBC (Giuli et al., 2019). Interestingly, by specifying the PAM-50 subtype, we agree with the literature trend of increasing NOTCH1 expression. The level of the expression was slightly higher than in normal tissue, although the observation was made only in basal-like BC (Table 3). Additionally, among ligands, DLL3 demonstrated significantly higher expression in comparison with other subtypes (logFC = 4.04 vs. 1.32, 0.55, and 1.03 in basal-like, HER2-enriched, luminal A and B BC, respectively; Table 3).

**Table 3:** Summary statistics on Notch core components including logFC according to the PAM50 subtypes of BC.

	**logFC**			
	**Basal-like**	**HER2-enriched**	**Luminal A**	**Luminal B**
*ADAM10*	−0.45	−0.12	−0.1	−0.19
*ADAM17*	0.28	−0.06	−0.27	−0.27
*APH1A*	1.04	0.7	0.7	0.77
*APH1B*	−1.73	−1.31	−0.12	−0.58
*ATXN1*	−0.63	−1.06	−0.89	−1.12
*ATXN1L*	−0.55	0.07	0.08	−0.16
*DLK1*	−6.99	−5.63	−7.16	–
*DLL1*	−1.56	−1.35	−0.95	−1.99
*DLL3*	4.04	1.32	0.55	1.03
*DLL4*	−1.07	−0.54	−0.69	−1.01
*DTX1*	−1.95	−2.39	−2.08	−3.1
*DTX2*	0.8	0.91	0.52	0.33
*DTX3*	2.01	1.95	2.12	2.23
*DTX3L*	−2.11	−1.79	−1.2	−1.35
*DTX4*	−0.13	0.07	−1.1	−1.12
*DVL1*	0.54	0.11	0.25	0.19
*DVL2*	−0.01	−0.47	−0.28	−0.22
*DVL3*	0.74	0.36	0.3	0.4
*EP300*	−0.33	−0.24	−0.21	−0.23
*HDAC1*	0.51	0.19	0.31	0.49
*HDAC2*	1.11	0.85	0.17	0.29
*HES1*	−0.42	0.04	−0.14	−0.23
*HES4*	1.16	1.34	0.99	0.85
*HES5*	−0.75	−0.24	0.03	−0.5
*HEY1*	−1.32	−1.07	−0.77	−1.14
*HEY2*	−1.84	−2.33	−1.85	−2.5
*HEYL*	0.17	0.81	1.14	0.66
*JAG1*	−0.68	−1.03	−0.52	−1.06
*JAG2*	−0.56	−1.23	−0.97	−1.13
*KAT2A*	0.17	−0.57	−0.06	0.32
*KAT2B*	−1.53	−1.44	−1.35	−1.38
*LFNG*	−1.42	1.03	0.62	0.64
*MAML1*	−0.08	−0.17	0.03	0.03
*MAML2*	−1.13	−2.73	−2.46	−3.23
*MAML3*	−1.45	−0.81	−0.33	−0.6
*MFNG*	−1.54	−1.7	−1.34	−1.81
*NCOR1*	−0.57	−0.6	−0.3	−0.23
*NCOR2*	−0.16	−0.21	0.11	0.08
*NCSTN*	0.57	0.24	0.44	0.36
*NOTCH1*	0.16	−0.81	−0.9	−1.03
*NOTCH2*	−0.34	−0.54	−0.32	−0.7
*NOTCH3*	0.1	0.11	0.02	−0.24
*NOTCH4*	−1.96	−1.7	−1.43	−1.87
*NUMB*	−0.55	−0.28	−0.29	−0.4
*NUMBL*	0.3	−0.4	−0.23	−0.23
*PSEN1*	−0.19	0.31	0.33	0.23
*PSEN2*	0.49	0.54	0.78	0.84
*PSENEN*	0.9	1.09	1.1	1
*PTCRA*	1.05	2.1	1.47	1.31
*RBPJ*	–	–	–	–
*RBPJL*	–	–	–	–
*RFNG*	0.11	−0.25	0.05	0.06
*SNW1*	0.06	0.03	0.06	0.09

To date, the significance of the essential modulators of Notch receptors and ligands is often being omitted in specific cancer types, thus offering limited insight into Notch signaling during carcinogenesis. As a brief recap, the intramembrane activation of the Notch signaling occurs in a cascade of successive cleavage events: SI proteolysis performed by Fringe (Lfng, Mfng, and Rfng), SII proteolysis performed by TACE (Adam17), and SIII proteolysis performed by γ-secretase complex (Psenen, Psen1, Psen2, Ncstn, Aph1a, and Aph1b).

Of the Fringe family, Lfng was recognized as a tumor suppressor. Zhang et al. revealed that mammary-specific deletion of *LFNG* induced the origins of basal-like and claudin-low breast tumors accompanied by the accumulation of NICD followed by an increase in the expression of Notch targets and amplification of the *Met/Caveolin* locus, hence facilitating Jag/Notch signaling to promote basal-like BC (Xu et al., 2012). Similarly, Lfng was also shown as suppressive in prostate cancer (Zhang et al., 2014b) and pancreatic cancer (Zhang J. et al., 2016; Zhang S. et al., 2016). In turn, *MFNG* was highly expressed in claudin-low BC and its silencing reduced migratory potential and tumorsphere formation as well as decreased the stem-like population of cells *in vivo* (Zhang et al., 2015). Our study showed the overexpression of *LFNG* in BC and EC in comparison with corresponding normal tissues. *MFNG* was strongly decreased with the lowest expression in CC vs. normal tissue. *RFNG* was similarly lower in CC than in its normal compartment with no significant alterations in BC and EC (Table 2). Consistent with Zhang's research, we observed downregulation of *LFNG* in basal-like BC, despite overexpression in the HER-2 enriched and luminal BC subtypes in comparison with normal breast tissue (Table 3). Importantly, we did not observe any association of either *LFNG* or *MFNG* with OS (Table 4). Regarding DFS, lowered *LFNG* was associated with better prognosis in BC (cutp: HR = 1.92, *p* = 0.039; maxstat: HR = 2.03, *p* = 0.022), as opposed to OV (cutp: HR = 0.681, *p* = 0.0352; maxstat: HR = 0.674, *p* = 0.0341) and EC (cutp: HR = 0.325, *p* = 0.00254; maxstat: HR = 0.364, *p* = 0.00884; Table 5). Heightened *MFNG* predicted better outcomes in BC (cutp: HR = 0.353, *p* = 0.0143; maxstat: HR = 0.337, *p* = 0.0101), CC (cutp: HR = 0.215, *p* = 0.00556; maxstat: HR = 0.296, *p* = 0.0165), and EC (cutp: HR = 0.347, *p* = 0.0432; maxstat: HR = 0.345, *p* = 0.0419) in contrast to OV (HR = 1.63, *p* = 0.00173; Table 5). Surprisingly, our analysis revealed suppressive character of *RFNG*, whose higher expression was associated with more favorable OS in BC (cutp: HR = 0.221, *p* = 0.00146; maxstat: HR = 0.242, *p* = 0.00299), CC (maxstat: HR = 0.595, *p* = 0.0457), and OV (cutp: HR = 0.633, *p* = 0.0366; maxstat: HR = 0.624, *p* = 0.0341; Table 4), and more favorable DFS in BC (HR = 0.341, *p* = 0.011) and EC (HR = 0.412, *p* = 0.0204; Table 5).

**Table 4:** Summary table of OS analysis.

	**Cutp**				**Maxstat**			
	**BRCA**	**CESC**	**OV**	**UCEC**	**BRCA**	**CESC**	**OV**	**UCEC**
*ADAM10*	2.02*	2.05*	1.47*		2.16**	1.98*	1.55*	
*ADAM17*		2.22**				2.46***		
*APH1A*	0.359***		0.654**	2.53*	0.349***		0.65**	2.53*
*APH1B*		2.44***	1.43*			2.45***	1.48*	
*ATXN1*			0.607*	0.365**				0.411*
*ATXN1L*	1.91*		1.9**		2.42**			
*CIR1*		0.161***		0.423*	0.331*	0.512*		0.423*
*CREBBP*		0.59*	1.42*			0.59*	1.42*	
*CTBP1*	0.415**				0.357**			
*CTBP2*	0.482**			0.41**	0.451**			0.399**
*DLK1*		2.36*				2.52**		
*DLL1*		2.25*	1.69**			1.67*	1.68**	
*DLL3*	0.593*	2.62*		2.29*				2.6**
*DLL4*		2.79***	0.71*			3.02***	0.725*	
*DTX1*	0.386*	0.441**			0.386*	0.437**		
*DTX2*		0.317***	1.45*			0.321***		
*DTX3*	0.21***	0.408*			0.44*		1.64*	<0.001*
*DTX3L*		0.494*				0.518*		
*DTX4*	0.461*				0.461*			
*DVL1*		2.83**	1.49*			2.83**		
*DVL2*	0.522*				0.522*			2.55**
*DVL3*				2.14*				2.14*
*EP300*								
*HDAC1*								
*HDAC2*	2.11**	1.74*	1.43*		2.08**	1.75*	1.43*	2.7**
*HES1*								1.98^(0.059)^
*HES4*	0.402***	1.99*	0.702*	0.325**	0.402***	2.13**	0.702*	0.374**
*HES5*	1.86*	0.525*			2.35**	0.323*		
*HEY1*								2.03^(0.0539)^
*HEY2*	0.56*		0.723*		0.56*	2.54*	0.691*	
*HEYL*		2.27*				2.49**		
*JAG1*		1.73*				1.9*		
*JAG2*				2.17^(0.0522)^				2.16^(0.053)^
*KAT2A*	0.267*							
*KAT2B*	2.27**	0.524*			2.27**	0.542*	0.734*	0.44*
*LFNG*		0.569^(0.0556)^				0.569^(0.0556)^		
*MAML1*						1.94*		
*MAML2*	0.546*				0.546*			
*MAML3*	0.61^(0.0534)^	1.69*		2.94**	0.61^(0.0534)^			3.52***
*MFNG*		0.552*		0.32***	2.42^(0.0514)^	0.529*	1.35^(0.059)^	0.35**
*NCOR1*	2.52*				2.49*			
*NCOR2*	0.52*	2.34**		0.426*	0.459*	2.51***		0.419*
*NCSTN*		3.29**		0.175^(0.0522)^		4.92**		0.169*
*NOTCH1*	1.66*	1.73^(0.0577)^		2.22*	1.66*	1.67*		3.56**
*NOTCH2*			1.43*	2.42*			1.44*	7.31*
*NOTCH3*				2.6**				2.6**
*NOTCH4*	1.96^(0.0528)^	1.92*				1.96*	0.602*	
*NUMB*			1.55**	0.269***			1.54**	
*NUMBL*	0.59*	0.253*	1.35^(0.0544)^		0.548*	0.253*		
*PSEN1*		1.88*		0.264*		2.14**		0.343**
*PSEN2*	0.576*	1.89*	0.407**			1.99**	0.453***	0.35*
*PSENEN*	0.311**			0.411*	0.311**			0.287***
*PTCRA*		0.506*				0.542*		
*RBPJ*		0.489**		0.483*		0.478*		0.46*
*RBPJL*				3.13**				2.43**
*RFNG*	0.221**		0.633*		0.242**	0.595*	0.624*	
*SNW1*			0.485*				0.532*	

*Value represents HR with statistical significance, and the color indicates expression level correlating with favorable prognosis: red—higher expression favorable, blue—lower expression favorable (level of the expression is considered relative to the determined cutpoint)*.

* *p < 0.05*.

** *p < 0.01*.

*** *p < 0.001*.

**Table 5:** Summary table of DFS analysis.

	**Cutp**				**Maxstat**			
	**BRCA**	**CESC**	**OV**	**UCEC**	**BRCA**	**CESC**	**OV**	**UCEC**
*ADAM10*			1.67**	2.84**			1.67**	3.91***
*ADAM17*	0.514*			3.3***	0.511*	2.5*		3.04**
*APH1A*		2.86**		2.44*		2.86**		2.44*
*APH1B*	0.198**	0.398*		3.05**	0.198**		0.559*	3.01**
*ATXN1*	1.94*				3.36*			
*ATXN1L*								
*CIR1*			1.54*				1.61*	
*CREBBP*			1.62**	2.34*		2.33*	1.62**	2.66**
*CTBP1*	2.34*				2.96*		1.58*	
*CTBP2*	0.488*	<0.001*		3.16**	0.485*	<0.001*		3.73***
*DLK1*			0.653*					
*DLL1*			1.46^(0.0518)^	0.396**				0.388*
*DLL3*	1.78^(0.0573)^	2.18*	2.18^(0.0546)^	3.15**		2.18*		2.95**
*DLL4*	2.86*	0.46^(0.056)^			2.86*		0.661*	
*DTX1*	0.483*		0.686*				0.652*	
*DTX2*	2.13*	0.17***		0.382*	2.29*	0.17***		0.268***
*DTX3*	0.207***				0.35**			
*DTX3L*		0.409*				0.391**		
*DTX4*								
*DVL1*	0.515*	2.36*	1.46*			2.52*		
*DVL2*	0.501*							
*DVL3*	2.06*			2.43*	2.05*			2.74**
*EP300*								
*HDAC1*		0.473*				0.473*		
*HDAC2*	2.35**				2.31**			
*HES1*		2.2*		0.434*		2.63**	0.6*	0.434*
*HES4*			0.643**	0.491*			0.615**	0.329**
*HES5*	1.82*		0.688^(0.0508)^	0.471^(0.0522)^	2.1*			
*HEY1*	2.85*		0.665*		3.66**		0.568*	
*HEY2*			0.64**	0.458*		2.54*	0.64**	0.458*
*HEYL*	3.51*	3.11*			4.91*	4.31**		
*JAG1*	100>^*^	3.23**				3.23**	0.612*	
*JAG2*			0.675*	0.193***				0.215***
*KAT2A*	<0.001*					2.24*		
*KAT2B*		0.426*						
*LFNG*	1.92*		0.681*	0.325**	2.03*	2.21^(0.0513)^	0.674*	0.364**
*MAML1*				2.11*				2.4*
*MAML2*			1.35^(0.0562)^			<0.001*		
*MAML3*		100>^*^			2.26*	100>^*^		
*MFNG*	0.353*	0.215**	1.63**	0.347*	0.337*	0.296*	1.63**	0.345*
*NCOR1*								
*NCOR2*		0.323**		2.23*		0.368**	2.11^(0.0533)^	
*NCSTN*	5.59^(0.0553)^	2.25*				2.41*		0.434*
*NOTCH1*	3.14**			0.376**	3.13**		0.732^(0.05)^	0.36*
*NOTCH2*			1.37*				1.6**	
*NOTCH3*				2.71**				2.71**
*NOTCH4*	5.37**	2.6*			5.24**	2.75*		
*NUMB*	0.46*				0.503*		1.7*	
*NUMBL*	0.433*	3.88***		2.91*	0.476**	3.76***		3.52**
*PSEN1*	0.531*		1.4*	0.368**	0.56^(0.0569)^	0.448*	1.41*	0.336**
*PSEN2*	0.525*		0.632**				0.449**	
*PSENEN*						0.523*		2.16*
*PTCRA*	2.7**	0.337*	0.71*		2.7**	<0.001*	0.675*	
*RBPJ*	2.97***		0.71*		3.22***		0.675*	
*RBPJL*				2.72**		2.68*		3.17**
*RFNG*	0.341*			0.412*	0.341*			0.412*
*SNW1*								2.16*

*Value represents HR with statistical significance, and the color indicates expression level correlating with favorable prognosis: red—higher expression favorable, blue—lower expression favorable (level of the expression is considered relative to the determined cutpoint)*.

* *p < 0.05*.

** *p < 0.01*.

*** *p < 0.001*.

Multiple studies reported oncogenic activity of *ADAM17* and its overexpression promoting tumorigenesis and disease progression in various cancers including, among others, BC (Shen et al., 2016) and TNBC (Caiazza et al., 2015). *ADAM17* has also been proposed as a therapeutic target, especially in OV, to enhance the efficiency of platinum-based therapies and diminish the acquisition of secondary chemoresistance (Hedemann et al., 2018). In our study, *ADAM17* was decreased in BC compared to the breast normal compartment; however, specifically in basal-like BC, the expression was higher than in normal tissue (Table 3). Similarly, the expression was higher in CC vs. normal tissue (Table 2). The survival analysis confirmed the oncogenic character of *ADAM17* as the lowered expression predicted better OS in CC (cutp: HR = 2.22, *p* = 0.00373; maxstat: HR = 2.46, *p* = 0.001; Table 4). Regarding disease recurrence, higher *ADAM17* was associated with better outcomes in BC (HR = 0.198, *p* = 0.00275), whereas lowered expression was more favorable in CC (maxstat: HR = 2.5, *p* = 0.0383) and EC (cutp: HR = 3.3, *p* = 0.000521; maxstat: HR = 3.04, *p* = 0.00326; Table 5). In addition, among CC patients, we were able to distinguish third survival group differing in prognosis regarding the drop in *ADAM17* expression, indicating an improving prognosis (Table 6).

**Table 6:** Summary table of OS analysis regarding the three groups.

**Gene**	**Tumor**	**Comparison**	HR*
*HES5*	BRCA	Low vs. high	0.397**
		Low vs. medium	0.744
		Medium vs. high	0.507^(0.0529)^
*ADAM17*	CESC	Low vs. high	0.146***
		Low vs. medium	0.377*
		Medium vs. high	0.492*
*DLL1*		Low vs. high	0.293**
		Low vs. medium	0.73
		Medium vs. high	0.456*
*HES4*		Low vs. high	0.141***
		Low vs. medium	0.428*
		Medium vs. high	0.479*
*HES5*		Low vs. high	8.17**
		Low vs. medium	2.21^(0.0627)^
		Medium vs. high	2.93^(0.0581)^
*JAG1*		Low vs. high	0.39*
		Low vs. medium	0.661
		Medium vs. high	0.392*
*NOTCH1*		Low vs. high	0.51*
		Low vs. medium	0.679
		Medium vs. high	0.728
*NOTCH1*	UCEC	Low vs. high	0.237**
		Low vs. medium	0.523
		Medium vs. high	0.348^(0.0686)^
*NOTCH2*		Low vs. high	0.105*
		Low vs. medium	0.152*
		Medium vs. high	0.422*

*Value represents HR with statistical significance, and the color indicates expression level correlating with unfavorable prognosis: red—higher expression unfavorable, blue—lower expression unfavorable (level of the expression is considered relative to the determined cutpoint)*.

* *p < 0.05*.

** *p < 0.01*.

*** *p < 0.001*.

Among the remaining Notch regulators such as Deltex (*DTX1, DTX2, DTX3, DTX3L*, and *DTX4*), Numb (*NUMB* and *NUMBL*), and Dvl (*DVL1, DVL2*, and *DVL3*), we observed diversified effects in tumors of different sites in the female tract. However, to date, the literature devoted to their involvement in the carcinogenesis of the female tract organs is very limited, and for that reason, the results of our analysis have been presented in Tables 2–5 and have not been hereby discussed.

### Signaling by the Core—Signal Transduction and HES/HEY TFs

Activation of the Notch signaling leads to the formation of an effector complex (CSL) consisting of RBP-J, specific co-activators [MAML family and histone acetyltransferases (HATs)], and co-repressors (CtBP, histone deacetylases HDAC, CIR, and ATXN1/L) to consequently derepress or activate promoters of *HES/HEY* genes. RBP-J is thus the most essential primary effector of the Notch signaling prompting to analyze its alterations, especially in tumorigenesis. The model systems of human BC revealed depletion of *RBPJ* resulting in increased cell survival and enhanced tumorigenicity due to the signal relegation to MYC and NF-κB (Kulic et al., 2015); however, it was reported as generally enhancing tumor growth and metastases in *Drosophila* (Liefke et al., 2010). In the present study, we observed that higher expression of *RBPJ* correlated with improved survival or more favorable disease-free prognosis in CC, OV, and EC, although surprisingly lowered levels were associated with better DFS in BC (Tables 4, 5). Among RBP-J regulators, *MAML1*, the main Notch co-activator, has been linked with the EMT and BC progression. In the knockdown studies involving MCF7 and MDA-MB-231 BC cell lines, it was concluded that *MAML1* may be considered as a negative regulator of EMT, thus limiting the rate of metastasis and BC relapse. Nevertheless, the relevance of the other regulators has not been elucidated. Our analyses indicated the downregulation of MAML family in BC, CC, and EC in comparison with their corresponding normal compartments (Tables 2, 3); nonetheless, the effects of expression of specific MAML on either OS or DFS varied (Tables 4, 5). Among histone acetyltransferases, *CREBBP* and *EP300*, we observed an increased frequency of mutations and CNVs, especially in CC and EC (*CREBBP*: 7.2% mutated cases and 1.7% CNV in CC, 8.9% mutated cases and 0.9% CNV in CC; *EP300*: 10.8% mutated cases and 2.4% CNV in CC, 8.9% mutated cases and 1.7% CNV in EC; Table 2). Moreover, as shown in Tables 4, 5, *ATXN1, CREBBP, CTBP1/2, KAT2A/B, HDAC1/2, CIR1*, or *SNW1* significantly differentiated patient outcomes reflecting the oncogenic or suppressive character of specific genes, which, to our best knowledge, is the first study describing their relevance in the female tract malignancies.

The Notch signaling ultimately leads to activation of Notch-specific TFs of the HES/HEY family triggering the cellular response through their downstream target effectors associated with processes such as apoptosis, proliferation, EMT, etc. Recently, Hes1 was shown in the maintenance of breast CSCs, metastasis, and halting the drug-induced apoptosis (Liu et al., 2015). Besides, the overactivation of Hes1 and Hes5 was observed among CC cases compared to CIN or normal cervical epithelia and furthermore correlated with poor prognosis of early-stage CC patients (Liu et al., 2007) that likely affected cell differentiation and promoted survival of CSCs through Notch–Hash interactions (Liu et al., 2010). We observed downregulation of *HES1* and *HES5* in BC, with the lowest values in basal-like BC, in comparison with normal breast tissue, whereas both were overexpressed in CC or EC vs. corresponding normal compartments. *HEY1*, apart from alterations of expression, was more frequently mutated in ~10% of BC and OV cases. On the other hand, *HEY2* was decreased in all of the tumors compared to the normal compartments, whereas *HEYL* levels dropped only in CC and EC (Tables 2, 3). We also observed associations of *HES* and *HEY* genes with patients' survival and tumor recurrence (Tables 4, 5), which may originate from differential activation patterns of the downstream effectors associated with the most essential biological processes frequently deregulated during carcinogenesis.

### Signaling by Notch—The Downstream Effects

**Figure 4:**
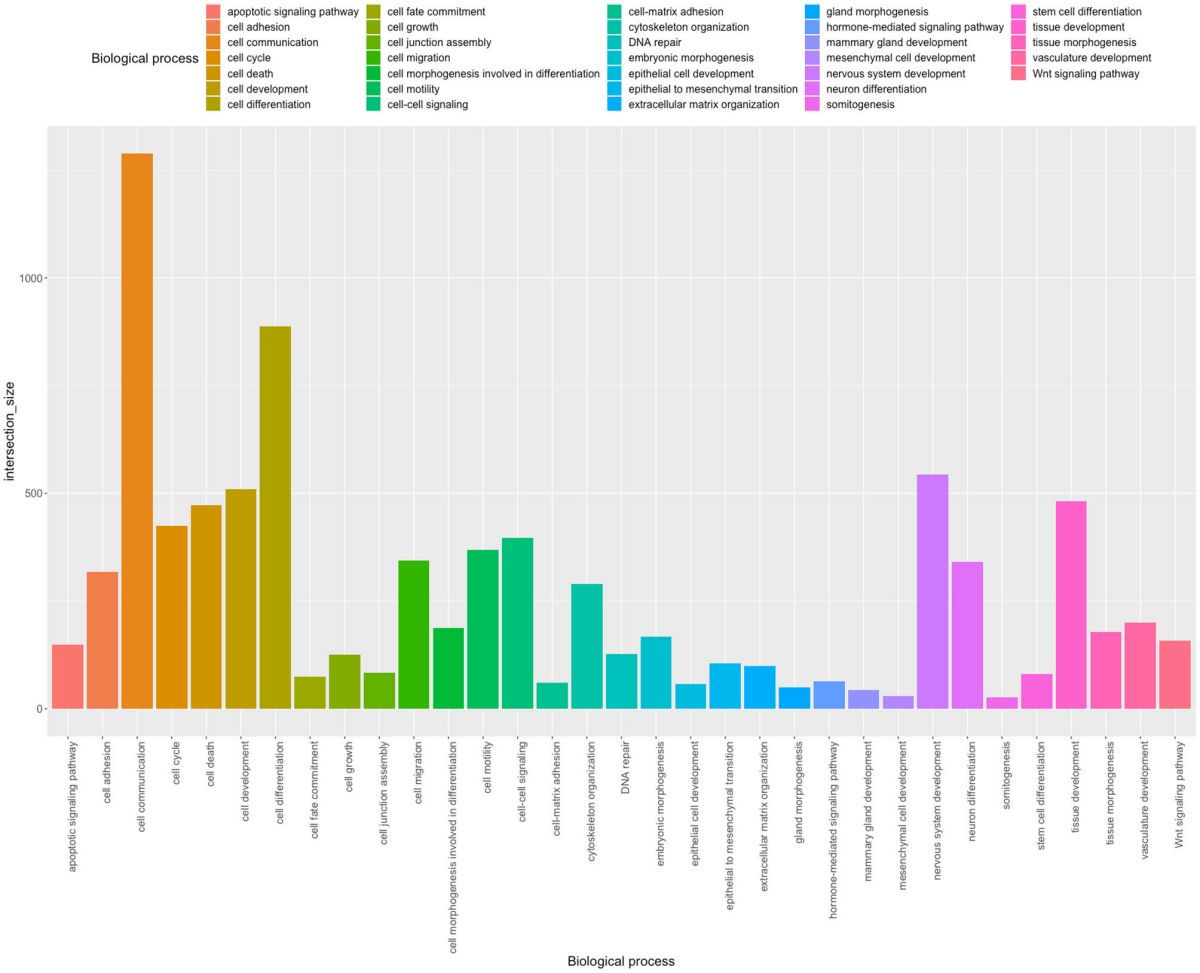
Functional annotation of HES and HEY downstream targets.

Yet, the Notch roles have been well-established in embryogenesis and adult life. Numerous research demonstrated how Notch orchestrates two principal processes such as cell fate determination and maintenance of SCs (e.g., Fiuza and Arias, 2007; Andersson et al., 2011; Hori et al., 2013; Siebel and Lendahl, 2017). Both great cellular machinery entail and initiate an effect of downstream dissemination of Notch signals through *HES* and *HEY* TFs. An excellent illustration of the above is a number of Notch downstream targets that we have identified and employed in this study through the GTRD database of ChIP-seq-identified TF binding sites. Analysis of HES1, HES5, HEY1, HEY2, and HEYL targets resulted in a total of 3,054 different genes. To provide a wider understanding of the mechanisms regulated downstream to Notch signaling, independently of tumorigenesis, we performed over-representation analysis (ORA) of biological terms among identified HES/HEY targets. The most essential processes have been shown in Figure 4. Beside broadly considered development, the most pivotal mechanisms are attributed to cellular death (apoptosis), DNA repair, proliferation, differentiation, cell cycle, and tissue architecture/remodeling-associated processes (adhesion, motility, ECM interactions, and EMT). However, the significance of these effects in the context of carcinogenesis and disease progression driven by Notch signaling is bypassed, and to date, only limited evidence of very specific context can be found in the literature, thus indicating lack of the comprehensive view of that area. Thereby, beyond the relevance of the Notch core components on the carcinogenesis of the female tract, we additionally included the second dimension of our considerations, which is the analysis and review of the major biological processes associated with tumorigenesis and/or progression that are targeted by HES/HEY downstream to Notch signaling among BC, CC, OV, and EC.

**Figure 5:**
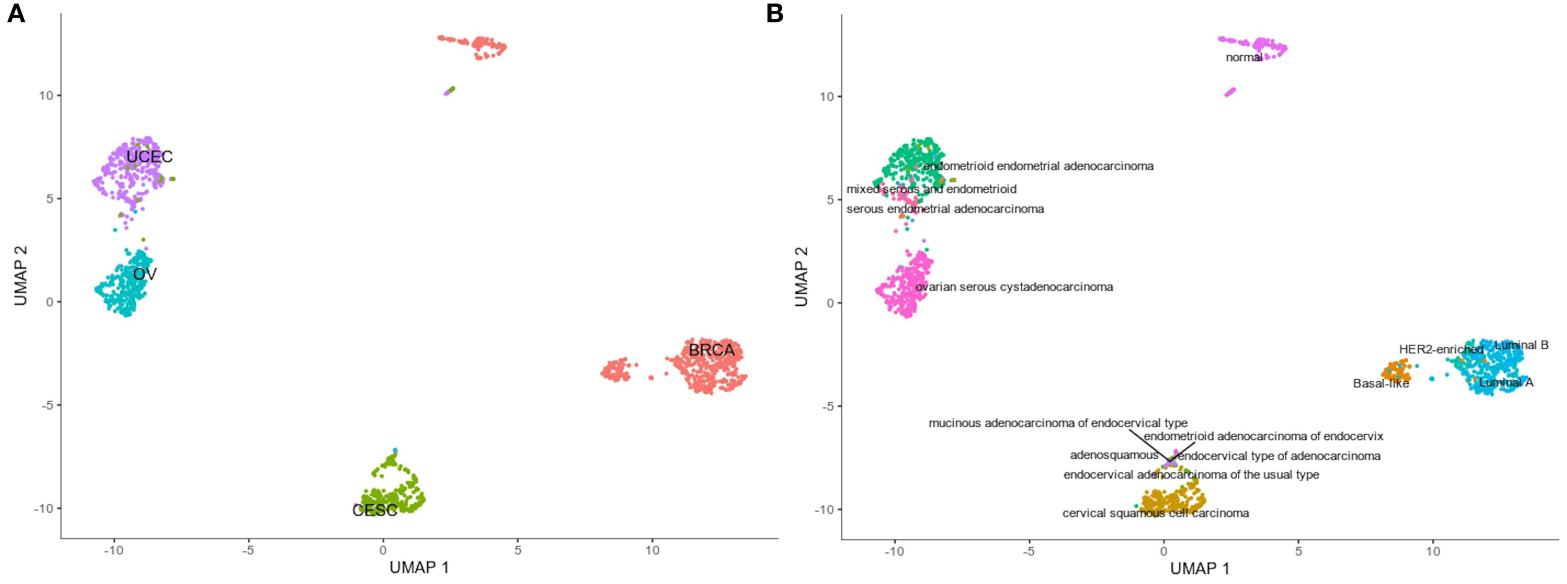
Spatial profiling of BC, CC, OV, and EC accompanied by normal tissues regarding the expression of downstream targets of Notch signaling. **(A)** shows the distribution of the cancer types accompanied by the normal tissues and the **(B)** specifies subtypes of the tumors with differentiated basal-like BC and normal tissues, independently of origin.

The abundance of biological processes that occur distinctly dependently of the tissue type and genetic alterations, especially during carcinogenesis, and are driven by Notch as a distant effect of the core signaling, has found its reflection in the grouping of BC, CC, OV, and EC within UMAP spaces regarding the resultant expression of the 3,054 downstream targets of Notch. These results indicated a common biological response posterior to Notch activation among the normal tissues, regardless of the diverse influence of hormonal regulation. If so, the question is how are the downstream effects of Notch signaling differentiated by the pathway in the tumors of the female tract. These malignancies vary regarding the clinical course of the disease as well as their biology, and these differences tend to originate from differential Notch signaling as a superior regulator. The findings were similar to the previous UMAP clustering concerning the expression of the core components, although of greater contrasts between the groups. In particular, BC and CC were the most distinct clusters of samples, as they were separated along with UMAP1 and UMAP2 spaces. OV and EC formed more similar clusters regarding UMAP1, of more different characteristics than to the core signaling along UMAP2. Moreover, basal-like BC formed a very distinct entity of samples, the same as the normal tissues independently of the primary origin (Figure 5).

**Figure 6:**
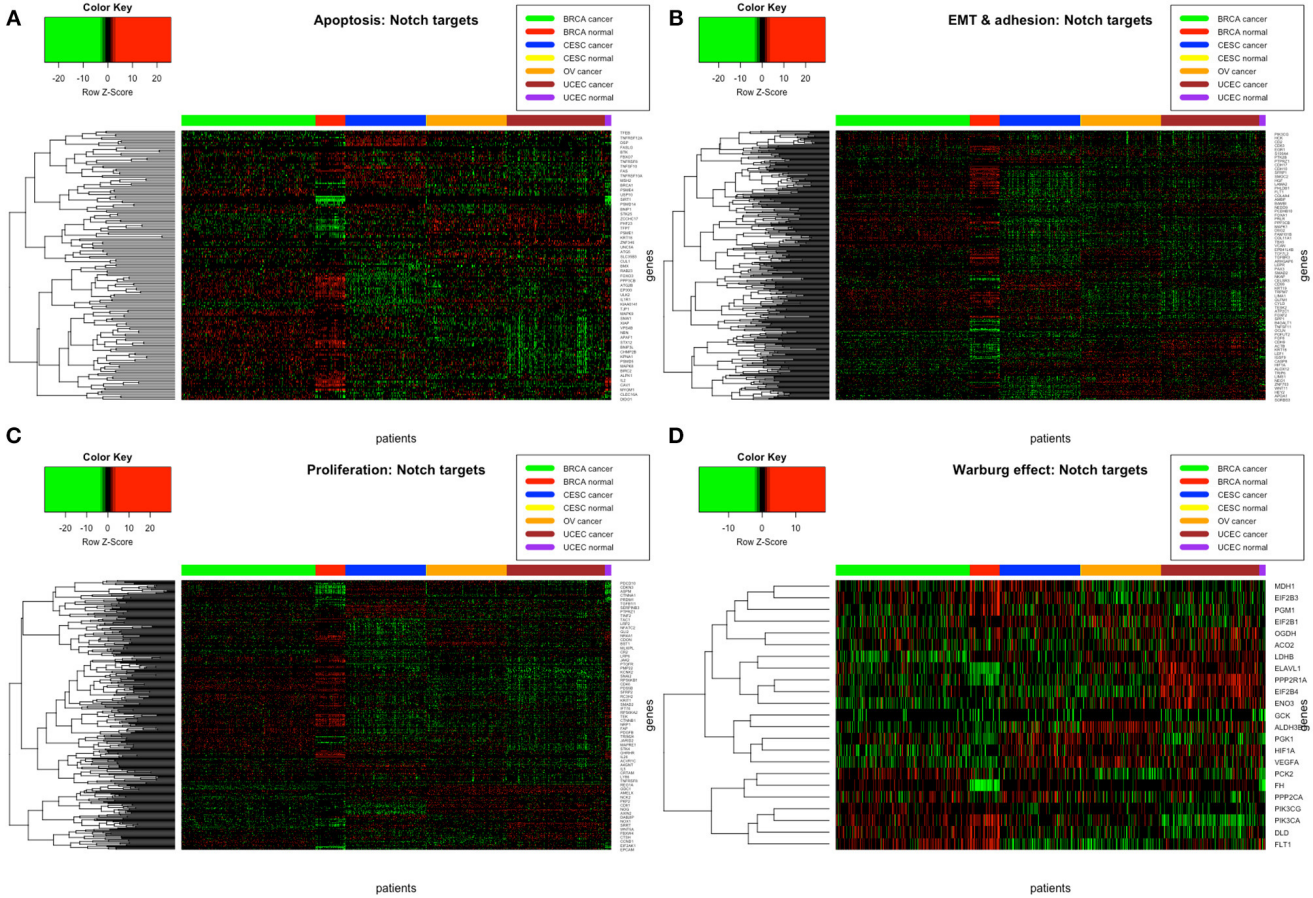
The profiles of expression of the Notch downstream effectors associated with **(A)** apoptosis, **(B)** adhesion and EMT, **(C)** proliferation, and **(D)** cell energetics, i.e., Warburg effect.

According to the biological processes recognized among the Notch downstream targets, we further focused on sets of genes associated with the major mechanisms that are abrogated during carcinogenesis and progression, especially concerning the cancer hallmarks, such as apoptosis, adhesion and EMT, proliferation, and Warburg effect, and revealed the expression profiles reflecting distinct spatial partitioning of BC, CC, OV, EC, and corresponding normal tissues. Figure 6 presents heatmaps of predefined sets of specified ontology, which confirm the former findings and emphasize how pleiotropic are distant effects of Notch signaling and the significance of the pathway during the essential events of carcinogenesis followed by a progression of the disease, especially in the female tract organs.

Normal development is generally controlled by a balance between cell proliferation and apoptosis, although the tumorigenesis occurs not only due to uncontrolled proliferation, but simultaneously reduced apoptosis. This balance essentially determines the overall growth or regression of cancer in response to various factors such as chemo- and radiotherapy or hormonal treatments, which all act by inducing apoptosis. Thus, expression profiles of apoptosis and proliferation-associated genes allow delineating of the biology of the individual tumors that may be further exploited to clinical advantage. Observable deregulation affecting the efficacy of the apoptotic mechanism may also be considered as a potential cause of treatment failure. Recapping, Notch as an arbiter of cell fate is a superior regulator of both processes, as, depending on the context, it orchestrates rate of proliferation and apoptotic cell death (Miele and Osborne, 1999). To date, it has been established that increased rates of apoptosis are related to the tumors of advanced grades and the ER-negative cells of BC. Moreover, the tumors of more aggressive character showed increased apoptosis and proliferation as well as correlated with a worse prognosis. Besides, the mitotic activity index (MAI) was shown as a very strong prognostic factor associated with the tumor size and lymph node status (van Diest et al., 2004). The findings on CC, OV, and EC linked the resistance to chemo- and hormone therapies with the impaired apoptosis and shifted the balance toward uncontrolled proliferation. Nonetheless, it is worth emphasizing that high rates of proliferation accompanied by relatively high rates of apoptosis are a manifestation of at least partial persistence of the physiological control mechanisms over the tumorigenesis.

The EMT is a complex process of acquiring by the epithelial cell a mesenchymal phenotype through a cascade of biological events. During carcinogenesis, these changes involve loss of adhesion, remodeling of the cytoskeleton architecture, as well as altered cell polarization, detachment from the ECM, migration, and intra- and extravasation, ultimately leading to the formation of the metastasis. From a morphological point of view, the EMT is characterized by the epithelial dedifferentiation to the mesenchymal phenotype usually accompanied by a loss of E-cadherin followed by increased expression of N-cadherin, vimentin, and cellular proteases. Thereby, the EMT represents the transitory state in the disease progression from the organ confined to a metastatic spread. To date, the Notch pathway has been shown as a key factor in the promotion and regulation of the EMT. The major regulatory mechanism involves direct transcriptional activation of Snail expression, a crucial TF promoting the repression of E-cadherin (Kar et al., 2019). These alterations have been associated with progression, metastasis, and more aggressive clinical course of BC (De Francesco et al., 2018), CC (Rodrigues et al., 2019), OV (Huang et al., 2019), and EC (Makker and Goel, 2016), although the accompanying Notch overexpression was observed especially among the basal-like BC (Fedele et al., 2017). Notably, these findings focused on the core signaling omitting the distant effects of the Notch pathway regarding the adhesion and EMT-associated processes. Figure 6B demonstrates how the profiles of expression of the Notch target genes involved in adhesion and EMT are differentiated across the female tract malignancies, reflecting the diverse biology of each specific type of tumor irrespective of the simultaneous signaling by steroid hormones.

The aberrancies of the Notch pathway were also shown to contravene the cell energetics. The signals forcing cells to proliferate at an enormous rate affect the utilization of the nutrients, especially the glucose uptake. Of note, the cancer cells tend to alter their metabolism to satisfy the high demands for various compounds, thus ensuring further growth and invasion. This involves glycolytic shift resulting in increased glycolysis that occurs irrespective of the mitochondrial respiration, known as the Warburg effect (Warburg, 1925). Recently, it has been shown that even a weak impulse of Notch activity may elicit continuing metabolic changes resembling the Warburg effect (Slaninova et al., 2016). In the studies on BC, Martinez-Outschoorn et al. concluded that the acidic microenvironment resulting from the ongoing Warburg effect provides a favorable niche for generating the CSCs (Martinez-Outschoorn et al., 2011), the hypothesis that has been further extended in the study of Goodman and collaborators revealing company of the high Notch activity (Goodman, 2012). Besides promoting tumorigenesis, the metabolic changes associated with the Warburg effect were also shown to increase the drug resistance (Bhattacharya et al., 2016). On the other hand, many studies are more and more often emphasizing the importance of the tricarboxylic acid (TCA) cycle (commonly known as the Krebs cycle), the major route for oxidative phosphorylation, during the carcinogenesis (Anderson et al., 2018). We observed that the Notch downstream effectors associated with the energetics of the cell reflected various profiles of the Warburg effect among the gynecological malignancies. Remarkably, the expression of FH encoding fumarase, an enzyme that catalyzes the reversible hydration/dehydration of fumarate to malate during the TCA cycle, demonstrated opposite expression patterns in the tumors compared to the normal tissue (Figure 6D) and its deregulation complies with the reports (Eng et al., 2003).

**Figure 7:**
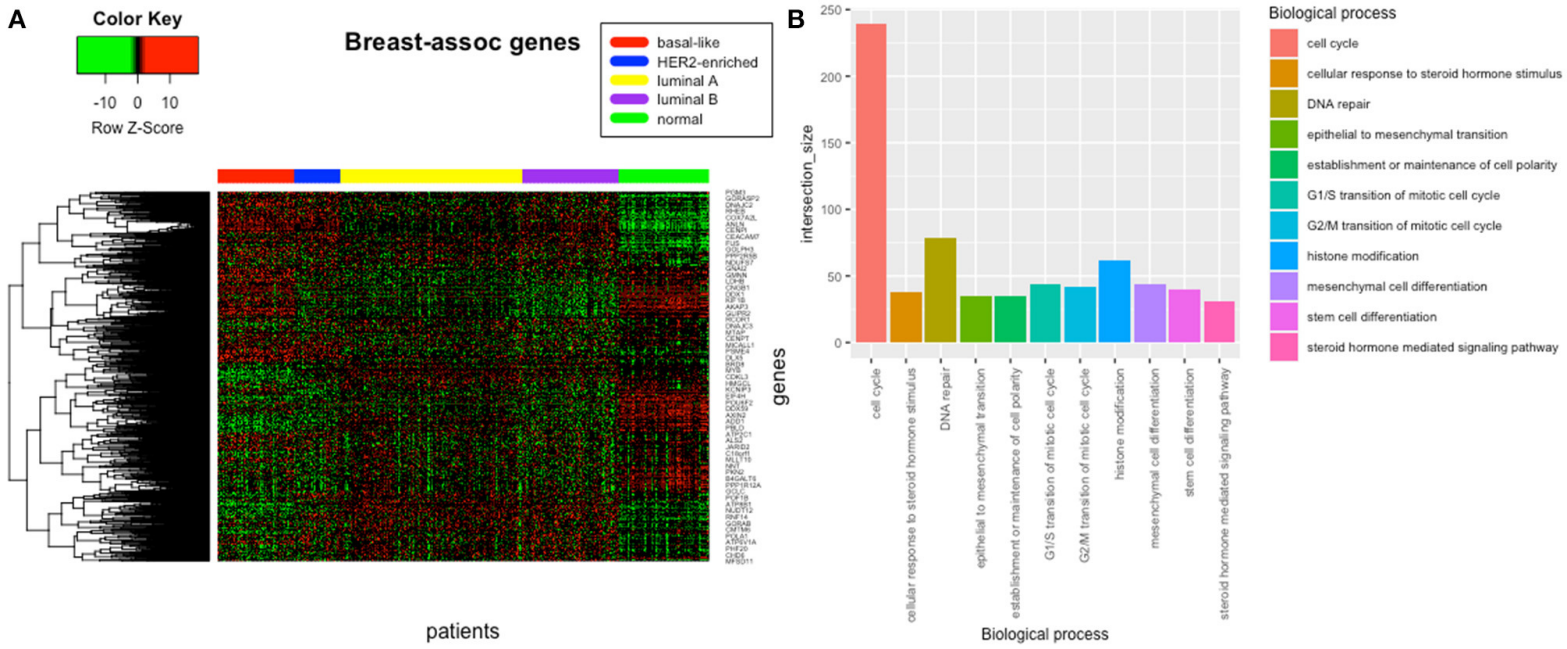
The Notch-derived profiles of expression characterizing subtypes of BC **(A)** with functional annotation of the biological processes **(B)**.

Finally, we performed WGCNA to elucidate the “otherness” factor of the cluster representing the basal-like BC in UMAP. The analysis revealed the module of 1,336 genes belonging to the Notch downstream targets that shared co-expression patterns in association with BC subtype. Beyond the major differences visible between cancer and normal breast tissue, basal-like was the most distinct, although similar to HER2-enriched. The latter also exhibited partial similarity to luminal subtypes, which were roughly homogeneous (Figure 7A). Regarding biological processes that these genes were involved in, we identified 190 terms that met the significance threshold. The most interesting were related to cell cycle, EMT, mesenchymal cell differentiation, DNA repair, G1/S and G2/M transition of the mitotic cell cycle, histone modification, SC differentiation, steroid hormone-mediated signaling pathway, and cellular response to steroid hormone signaling as well as establishment or maintenance of cell polarity, which very well represent differential biology and various clinical course of distinct BC subtypes (Figure 7B).

## Concluding Remarks

Notch pathway is one of the few major regulatory mechanisms during tissue development. Its deregulation affects normal proliferation and differentiation leading to aberrancies in tissue architecture and was also reported as an essential player in carcinogenesis and cancer progression including the female reproductive tract (breast, cervix, ovary, and uterine endometrium). Our analysis showed distinct gene expression profiles of Notch pathway members as well as their target genes.

Interestingly, though examined cancers show separated models of the Notch pathway and its targets, gene expression of all normal tissues is much more similar to each other than to its cancerous compartments, despite the different influence of hormone signaling, i.e., through estrogen. Such Notch-driven cancerous differentiation resulted in a case of opposite associations mainly with DFS and to less extent with OS that consequently reflects very distinct profiles of the target genes, including genes associated with cell proliferation and differentiation, energy metabolism, or the EMT. Expression of apoptotic genes differed among all cancers, but despite that, the most visible were differences between normal and cancerous tissues of the same type. Our analysis revealed that the Notch signaling pathway not only has a distinct influence on different female reproductive tract tissues but also demonstrated various roads of carcinogenesis. The differentiation of BC, CC, OV, and EC regarding the expression of the Notch core components visible in Figure 2 arose from the alterations in specific parts of the Notch pathway. BC and CC were closely related in the UMAP dimensions and, simultaneously, different from OV and EC forming another cluster of similar traits. By analogy, we observed the same trends in Notch-driven survival, which have been summarized in Table 7. It seems that the major differences in the Notch signaling originate from the different patterns of Notch activation through ligands of Delta and Serrate families. Despite all tumors showing the common profile of Notch receptors favoring lowered expression in terms of DFS, Delta and Serrate were similarly correlated with better prognosis in BC and CC, although different from OV and EC. However, the executors of Notch processing such as Fringe (SI cleavage), TACE (ADAM17 and SII cleavage), and γ-secretase complex (SIII cleavage) as well as the modulators (Dvl, Numb, and Deltex) seem to process the Notch signal similarly across the female tract tissues. The lowered activity of the *CSL* (RBP-J) effector complex was more favorable in BC, CC, and EC as opposed to OV with various profiles of co-activators and co-repressors, which could likely affect the signal transduction. Finally, the last members of the core signaling, *HES* and *HEY* TFs, reflected in trends the activation pattern of Delta and Serrate ligands and decreased expression in BC and CC, but the increased expression in OV and EC was associated with improved disease-free outcomes. The signaling map differing BC, CC, OV, and EC drawn by alterations in single genes may therefore serve as marker profiles resulting in specific clinical outcomes, which in turn originate from alterations of the downstream targets and associated biological processes. We based this comparative summary on the DFS as it seems to be less biased with the general condition of patients, coexisting diseases, and other clinical factors affecting OS; however, the trends in both analyses were largely consistent (Tables 4, 5). Quite simple signaling connections are functionally very differentiated; therefore, several mechanistic experiments are required to find and explain every specific change in expression of particular Notch pathway members. On the other hand, we may conclude that observed OS and DFS Notch pathway associations resulted from differential expression of target genes. This may direct a future analysis to search for new therapeutic targets based on specific Notch pathway profiles.

**Table 7:** The summary of Notch signaling differentiating BC, CC, OV, and EC.

**Group**	**Gene**	**BC**	**CC**	**OV**	**EC**
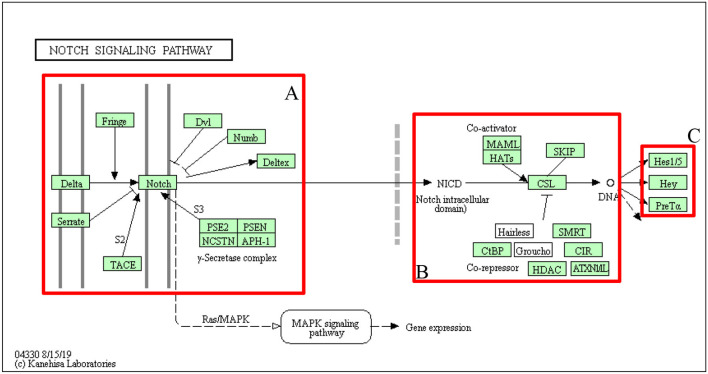					
**A: LIGANDS, RECEPTORS, AND MODULATORS**					
Delta	*DLL1*				↑
	*DLL3*		↓		↓
	*DLL4*	↑		↑	
Serrate	*JAG1*	↓	↓	↑	
	*JAG2*			↑	↑
Fringe	*LFNG*	↓		↑	↑
	*MFNG*	↑	↑	↓	↑
	*RFNG*	↑			↑
TACE	*ADAM17*	↑	↓		↓
Notch	*NOTCH1*	↓			↑
	*NOTCH2*			↓	
	*NOTCH3*				↓
	*NOTCH4*	↓	↓		
Dvl	*DVL1*	↑	↓	↓	
	*DVL2*	↑			
	*DVL3*	↓			↓
Numb	*NUMB*	↑		↓	
	*NUMBL*	↑	↓		↓
Deltex	*DTX1*	↑		↑	
	*DTX2*	↓	↑		↑
	*DTX3*	↑		↓	
	*DTX3L*		↑		
	*DTX4*				
γ-secretase complex	*PSENEN*		↑		↓
	*PSEN1*	↑		↓	↑
	*PSEN2*	↑		↑	
	*NCSTN*		↓		↑
	*APH1A*		↓		↓
	*APH1B*	↑	↑	↑	↓
**B: SIGNAL TRANSDUCTION**					
MAML	*MAML1*				↓
	*MAML2*		↑		
	*MAML3*	↓	↓		
HATs	*CREBBP*		↓	↓	↓
	*EP300*				
	*KAT2A*	↑		↓	
	*KAT2B*		↑		
SKIP	*SNW1*				↓
CSL	*RBPJL*		↓		↓
	*RBPJ*	↓		↑	
CtBP	*CTBP1*	↓		↓	
	*CTBP2*	↑	↑		↓
HDAC	*HDAC1*		↑		
	*HDAC2*	↓			
ATXN1/L	*ATXN1*	↓			
	*ATXN1L*				
CIR	*CIR1*			↓	
SMRT	*NCOR2*		↑		↓
**C: TRANSCRIPTION FACTORS**					
Hes1/5	*HES1*		↓	↑	↑
	*HES5*	↓			
Hey	*HEY1*	↓		↑	
	*HEY2*		↓	↑	↑
	*HEYL*	↓	↓		
PreTα	*PTCRA*	↓	↑	↑	

*The up arrow indicates the higher expression of a particular Notch member associated with favorable DFS prognosis and the down arrow indicates the lowered expression of a particular Notch member correlating with better DFS*.

## Materials and Methods

### Data Acquisition

The four TCGA cohorts including breast invasive carcinoma (BRCA; BC), cervical and endocervical cancers (CESC; CC), ovarian serous cystadenocarcinoma (OV), and uterine corpus endometrial carcinoma (UCEC; EC) were obtained from The Cancer Genome Atlas repositories through GDAC Firehose in the form of expression (RNAseqV2, level 3, RSEM normalized, data status of 28th Jan 2018) with corresponding clinical data. Patients that missed corresponding expression/clinical data were excluded from further analyses. Moreover, among BC, only female patients with available PAM50 classifier were analyzed. The summary and sizes of cohorts used in the study are shown in Table 8. Normal, paired solid tissues were additionally retrieved through R-dedicated package TCGA-Assembler (Wei et al., 2018).

**Table 8:** Sizes and classification of the cohorts used in the study.

	**No. of tumor samples**	**No. of matched normal samples**
BC:	505	
- Basal-like	97	113
- Luminal A	228	
- Luminal B	122	
- HER2-enriched	58	
CC:	304	
- Cervical squamous cell carcinoma	252	3
- Endocervical adenocarcinoma of the usual type	6	
- Adenosquamous	5	
- Endometrioid adenocarcinoma of endocervix	3	
- Endocervical type of adenocarcinoma	21	
- Mucinous adenocarcinoma of endocervical type	17	
OV	301	Not available
EC:	370	24
- Serous endometrial adenocarcinoma	57	
- Mixed serous and endometrioid endometrial adenocarcinoma	10	
- Endometrioid endometrial adenocarcinoma	303	

### Pathway-Associated Data

The scheme of a core signaling through Notch was accessed through the KEGG database (hsa04330) (Kanehisa and Goto, 2000; Kanehisa et al., 2019). The list of core members participating in the pathway was downloaded from MSigDB (Liberzon et al., 2015) according to the corresponding KEGG gene set. Detailed lists of genes involved in the Notch pathway are available in the GitHub repository (https://github.com/orzechmag/notchfemaletract).

Genes classified as downstream targets of the Notch pathway were identified through literature-based, well-known pathway-specific TFs. Subsequently, targets of the aforementioned executive TFs were identified through the GTRD database, which comprises a collection of ChiP-seq documented TF binding sites for human (Yevshin et al., 2017, 2019). Finally, Ensembl Gene ids were converted into Gene Symbols using the db2db tool from bioDBnet (Mudunuri et al., 2009). Detailed lists of target genes retrieved from GTRD are available in the GitHub repository (https://github.com/orzechmag/notchfemaletract).

### Pathway-Associated Global Profiling of Tumors

Population structure of BC, CC, OV, and EC accompanied by normal tissues was studied by applying the UMAP method, preceded by a PCA pre-processing step regarding the expression of core members of Notch as well as expression of its downstream effectors as two separate models through employing Monocle3 R package. Monocle3 is primarily dedicated to analyzing single-cell RNA-seq (scRNA-seq); however, except for trajectory-based analysis of tissue/lineage-specific differentiation, available tools (e.g., PCA pre-processing, UMAP, visualization tools, and suite for DGE analysis) are of general usage and therefore were applicable.

### Alterations of Pathway Core Members—DGE and Mutations

Basic alterations between cancerous and normal tissue were identified through the calculation of logarithmized fold change (logFC, i.e., log_2_FC) applied on members of the core of the Notch pathway. logFC was calculated between tumor and its matched normal tissue except OV as its corresponding normal tissue was not available in TCGA. Profiles of expression were shown by employing heatmaps generated with heatmap.2() function in R with the complete agglomeration method and Spearman distance metric. Moreover, mutations and copy number alterations (CNAs) occurring in pathway core genes accompanied by TP53 and DNA processing-associated enzymes such as DNMT1 (DNA methyltransferase 1) and HDAC1, HDAC2, HDAC4, and HDAC7 (histone deacetylases 1, 2, 4, and 7) were identified via cBioPortal (Cerami et al., 2012; Gao et al., 2013) among respective cohorts of BC, CC, OV, and EC.

### Notch-Specific Survival Analysis

Significance of core pathway members has been investigated in terms of clinical outcome; therefore, DFS and OS analyses were conducted. The analysis was performed with Evaluate Cutpoints system (Ogluszka et al., 2019) involving cutp, maxstat, and rolr algorithms of cutpoint determination in correlation with survival time and clinical outcome according to the following clinical parameters: “patient.person_neoplasm_cancer_status” and “patient.vital_status” as event indicator and “patient.days_to_last_followup” and “patient.days_to_death” as a time of observation for DFS and OS, respectively.

### Variability of Genes Associated With Specific Biological Processes Governed by the Notch Signaling

Among downstream effectors of Notch signaling, we identified sets of genes involved in major biological processes of indisputable relevance and contribution in carcinogenesis and disease progression such as apoptosis, adhesion (including EMT-related markers), proliferation, and Warburg effect. The sets of genes were created based on MSigDB collections of ontological terms (C5, BP: GO biological processes) and involved all terms that were widely associated with apoptosis, adhesion, EMT, proliferation, and cancer energetics (i.e., Warburg effect). Subsequently, each ontology was defined among downstream targets of the Notch signaling pathway resulting in the final sets of genes. Profiles of expression were presented in the form of heatmaps, analogously to the previous section. Gene sets of all ontological terms are available in the GitHub repository (https://github.com/orzechmag/notchfemaletract).

### Identification of the Basal-Like “Otherness” Factor Among the Subtypes of BC

Regarding the fact that basal-like BC formed a separate cluster in UMAP dimensions from the remaining BC subtypes, it may be considered as a distinct molecular characteristic with an inclination to become a separate disease entity (as in fact remains in line with literature reports). Thereby, we aimed to define the set of genes contributing to distinct characteristics of the basal-like subtype followed by functional annotation to define abrogated biological processes among downstream targets of the Notch pathway. Modules of genes sharing a common profile of expression with BC subtype were determined by applying weighted gene co-expression network analysis (WGCNA) with a soft-thresholding approach (β = 4) within the R environment (Langfelder and Horvath, 2008). The further analysis comprised functional annotation of genes concerning biological processes through g:Profiler (Raudvere et al., 2019) and visualization expression profiles in heatmaps, analogously to the previously described. The WGCNA R code is available in the GitHub repository (https://github.com/orzechmag/notchfemaletract).

## Data Availability Statement

Publicly available datasets were analyzed in this study. This data can be found here: GDAC Firehose (https://gdac.broadinstitute.org/) and GitHub (https://github.com/orzechmag/notchfemaletract).

## Author Contributions

AB was responsible for study design, data interpretation, and revision of the manuscript. DA and MO were responsible for data preparation, data analysis, study results, data interpretation, and writing of the paper. All authors contributed to the article and approved the submitted version.

## Conflict of Interest

The authors declare that the research was conducted in the absence of any commercial or financial relationships that could be construed as a potential conflict of interest.
